# Molecular mechanism of toxin neutralization in the HipBST toxin-antitoxin system of *Legionella pneumophila*

**DOI:** 10.1038/s41467-022-32049-x

**Published:** 2022-07-26

**Authors:** Xiangkai Zhen, Yongyu Wu, Jinli Ge, Jiaqi Fu, Le Ye, Niannian Lin, Zhijie Huang, Zihe Liu, Zhao-qing Luo, Jiazhang Qiu, Songying Ouyang

**Affiliations:** 1 Provincial University Key Laboratory of Cellular Stress Response and Metabolic Regulation, the Key Laboratory of Innate Immune Biology of Fujian Province, Biomedical Research Center of South China, Key Laboratory of OptoElectronic Science and Technology for Medicine of the Ministry of Education, College of Life Sciences, Fujian Normal University, 350117 Fuzhou, China; 2 State Key Laboratory for Zoonotic Diseases, College of Veterinary Medicine, Jilin University, Changchun, China; 3 Purdue Institute for Inflammation, Immunology and Infectious Disease and Department of Biological Sciences, Purdue University, West Lafayette, IN USA

**Keywords:** Enzyme mechanisms, Biochemistry, X-ray crystallography, Bacterial genetics

## Abstract

Toxin-antitoxin (TA) systems are ubiquitous genetic modules in bacteria and archaea. Here, we perform structural and biochemical characterization of the *Legionella pneumophila* effector Lpg2370, demonstrating that it is a Ser/Thr kinase. Together with two upstream genes, *lpg2370* constitutes the tripartite HipBST TA. Notably, the toxin Lpg2370 (HipT_Lp_) and the antitoxin Lpg2369 (HipS_Lp_) correspond to the C-terminus and N-terminus of HipA from HipBA TA, respectively. By determining crystal structures of autophosphorylated HipT_Lp_, its complex with AMP-PNP, and the structure of HipT_Lp_-HipS_Lp_ complex, we identify residues in HipT_Lp_ critical for ATP binding and those contributing to its interactions with HipS_Lp_. Structural analysis reveals that HipS_Lp_ binding induces a loop-to-helix shift in the P-loop of HipT_Lp_, leading to the blockage of ATP binding and inhibition of the kinase activity. These findings establish the *L*. *pneumophila* effector Lpg2370 as the HipBST TA toxin and elucidate the molecular basis for HipT neutralization in HipBST TA.

## Abstract (web summary)

Here, the authors demonstrate that the Legionella pneumophila T4SS effector protein Lpg2370 is a Ser/Thr kinase and a toxin of a tripartite HipBST toxin-antitoxin (TA) system. Structural data and biochemical analysis provide detailed insights into the toxin neutralization mechanism in the HipBST TA.

## Introduction

Toxin–antitoxin (TA) systems are bacterial and archaeal genetic modules enriched in mobile genetic elements and chromosomes that comprise two or more closely linked genes encoding a toxin protein and its cognate antitoxin^1^. Since the discovery that the ccdB/ccdA TA system maintains stable inheritance of the mini-F plasmid in *Escherichia coli*^2^, the biological roles of TA systems have been demonstrated to include maintaining stabilization and fitness of mobile genetic elements such as plasmids^3^ and protection against phages^4^. Toxins are stable enzymes (e.g., RNases and kinases) or other proteins (e.g., gyrase inhibitors and pore-like toxins) that, in the absence of cognate antitoxin, interfere with vital cellular processes such as DNA replication and protein translation^5^. Antitoxins are unstable proteins or RNAs that counteract toxins. Based on the antitoxin nature and toxin-neutralization mechanism, TA systems can be divided into types I–VIII^6,7^.

In type II TA systems such as HipBA modules, toxin neutralization depends on direct binding of a proteinaceous antitoxin^1,8^. HipA from HipBA TA module of the *E. coli* strain K-12 is a 440-amino acid (aa) Ser/Thr kinase that phosphorylates the tRNA^Glu^-bound glutamate-tRNA ligase GltX at Ser239, thereby inhibiting protein translation^9,10^. The growth arrest induced by *E*. *coli* HipA can be counteracted by HipB, a cro/C1-type helix–turn–helix (HTH) domain-containing protein^11^. The structures of HipBA modules from *E. coli* (HipBA_Ec_) and *Shewanella onesidensis* (HipBA_So_) reveal that both TA modules form a HipA_2_–HipB_2_ heterotetramer in which HipB binds far from the kinase catalytic center of HipA^12^. Such neutralization strategy differs from most of other type II TA systems, where antitoxins binding usually occludes the active site^13^ or mediate allosteric regulation of the toxin^14–16^.

Recent studies demonstrated that TA systems containing toxins homologous to the *E*. *coli* HipA are widely distributed in bacterial genomes^17^, suggesting the diversity of HipBA TA systems^17,18^. Among these TA systems, a tripartite system designated HipBST was recently identified and experimentally characterized in the enteropathogenic *E. coli* serotype O127:H6^18^. HipT, which serves as the toxin in the HipBST system, phosphorylates TrpS at Ser197, and its toxicity can be counteracted by the small protein encoded by the adjacent gene *hipS*^17^. Importantly, the toxin HipT and the antitoxin HipS of the HipBST system were found to correspond to the N-terminal subdomain 1 and the core kinase domain of the *E. coli* HipA, respectively. The third protein of the HipBST module, HipB, is analogous to HipB of the HipBA system and appears to enhance the neutralization effect of HipS by binding to an already formed HipT–HipS heterodimer^18^. Recently, a preprint study reported the structure of HipBST heterotrimer from *E*. *coli* serotype O127:H6 and concluded that ATP binding in HipT is prevented by comparing its structure in the heterotrimer to the available structures of *E. coli* HipBA complex^19^. However, the general mechanism for toxin neutralization in HipBST TA systems is not fully elucidated.

*Legionella pneumophila*, the causative agent of Legionnaires’ disease, extensively modifies host signal transduction pathway, especially the post-translational modifications such as ubiquitination and phosphorylation, by translocating hundreds of effectors into the host cell via the Dot/Icm system^20–23^. One such effector is the recently identified Lpg2370^24^, which was previously predicted to be an E3 ligase but has not been characterized^25^.

In this work, we find that Lpg2370 in fact shares sequence identity with the C-terminus of the *E. coli* K-12 HipA, the toxin of type II TA system HipBA, which is then confirmed by experimental validation and determining the crystal structure of autophosphorylated Lpg2370 (pLpg2370). Furthermore, gene locus analysis indicates that *lpg2370* is grouped with *lpg2369* and *lpg2368* into a tricistronic operon, which we proceed to characterize respectively as the toxin HipT_Lp_, the antitoxin HipS_Lp_, and HipB_Lp_, that constitute the tripartite TA system HipBST. We also determine high-resolution structures of the toxin pHipT_Lp_ in complex with the ATP analog AMP–PNP and the binary complex with the antitoxin HipS_Lp_ and identify key HipT_Lp_ residues involved in ATP binding and interactions with HipS_Lp_. Lastly, a comparison of the three structures determined in this study allowed us to propose the mechanism of toxin neutralization in the type II TA system HipBST.

## Results

### The *L. pneumophila* effector Lpg2370 is a Ser/Thr kinase

**Fig. 1:**
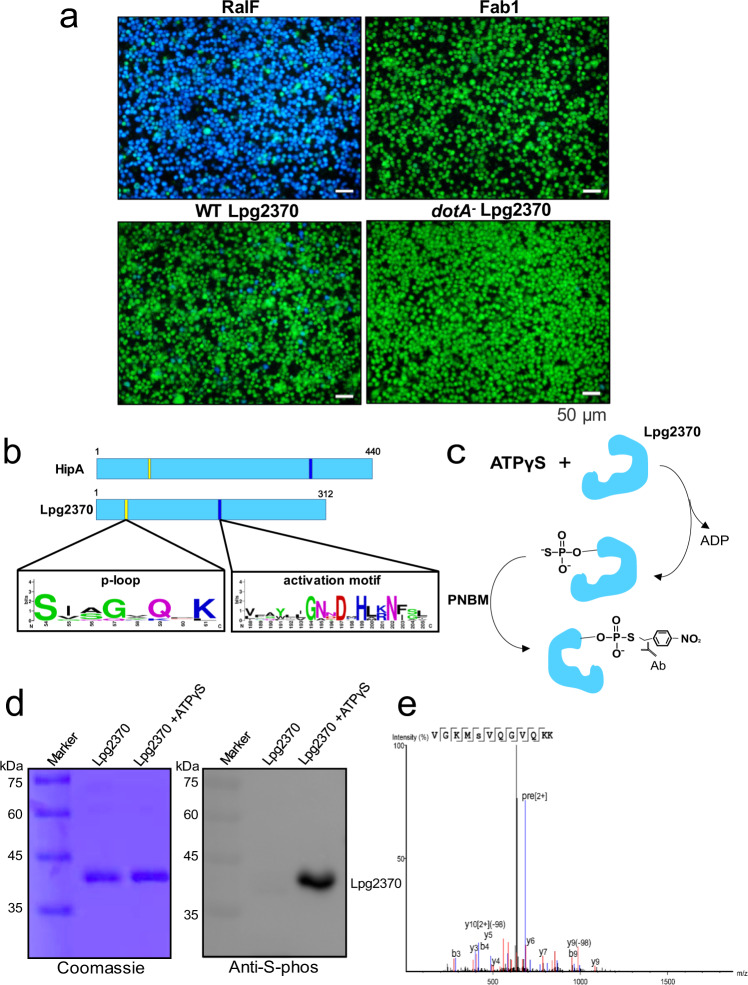
*L. pneumophila* effector Lpg2370 is a Ser/Thr kinase. **a** TEM-1 β-lactamase translocation assay demonstrates that Lpg2370 is a *L*. *pneumophila* effector protein. RAW264.7 cells were challenged with a T4SS-competent wild-type *L. pneumophila* strain Lp02 or the dotA-mutant deficient strain Lp03 carrying plasmids encoding TEM-1-RalF (positive control), TEM-1-FabI (negative control), or TEM-1- HipT_Lp_. T4SS-mediated translocation of the fusion proteins into host cells was assessed 2 h after infection by the CCF4-AM-based fluorescence resonance energy transfer assay, scale bars 50 μm. **b** Schematic of Lpg2370 and the Ser/Thr kinase HipA from *E. coli* K-12 (HipA_Ec_). Yellow and blue regions represent the approximate locations of the P-loop and activation motif of HipA, respectively, as well as the locations of corresponding sequences in Lpg2370. Sequence conservation of P-loop and the activation motif is presented as a weblog below the protein schematics. **c** Diagram depicting detection of thiophosphorylated Lpg2370 by thiophosphate labeling. PNBM: p-nitrobenzylmesylate. **d** Thiophosphate labeling assay with the purified 6×His-tagged Lpg2370. Thiophosphorylated Lpg2370 was visualized by immunoblotting. **e** Identification of the phosphorylated peptide by LC-MS/MS. The peptide SVQGVQK was observed at charge state 2^+^ in two forms differing by 70.97 Da in molecular mass.

We first verified whether Lpg2370 is translocated into host cells via the type IV secretion system (T4SS) Dot/Icm as an effector protein. To this end, we performed TEM-1 β-lactamase translocation assay by infecting RAW264.7 macrophages with the fusion protein-expressing *L. pneumophila* cells grown to post-exponential phase. Vectors expressing TEM-1-RaIF (positive control), TEM-1-FabI (negative control) or TEM-1-Lpg2370 fusion proteins were introduced into the T4SS-competent *L. pneumophila* strain Lp02 or the Dot/Icm-deficient strain Lp03, which were then assessed for the delivery of the β-lactamase fusions into the host macrophage cells by visual inspection under a fluorescence microscope. Cells infected by Lp02 cells expressing the TEM-RalF fusion protein emitted blue fluorescence, whereas infection with the TEM-FabI-expressing cells did not result in any emission of blue fluorescence by host cells (Fig. 1a). In addition, none of the fusion proteins were detectably translocated upon infection with the Dot/Icm-deficient strain Lp03 (Fig. 1a). Consistent with previous studies^24^, the Lpg2370-overexpressing *L. pneumophila* strain Lp02 can be secreted into host cells, though at very low translocation efficiencies, suggesting that Lpg2370 is indeed a *L*. *pneumophila* effector protein.

Although previous studies implied that Lpg2370 is an E3 ubiquitin ligase based on sequence similarity with the RING-type E3 ubiquitin ligase FANCL^25–27^, we repeated primary sequence analysis and did not find any significant similarity between the two proteins. However, we found notable sequence identity (~20%) between Lpg2370 and the residues 64–440 of *E*. *coli* HipA, an atypical Ser/Thr kinase from the *E. coli* K-12 strain (Supplementary Fig. 1)^28^. The results particularly indicated conservation of the sequences corresponding to the P-loop (RISVAGAQ), the signature motif responsible for ATP binding, and the catalytic loop, which contains the catalytic residue D310 required for the kinase activity of *E. coli* HipA^29–31^ (Fig. 1b). Moreover, comparing the Lpg2370 sequence to the NCBI database using basic local alignment search tool (BLAST) identified Lpg2370 as a HipA-like Ser/Thr kinase. These findings led us to hypothesize that Lpg2370 could be a kinase.

Kinases frequently undergo autophosphorylation on an invariable Ser or Thr residue in the P-loop^32^. For instance, autophosphorylation of Ser150 on the P-loop has been observed in HipA kinases from *E*. *coli* (HipA_Ec_) and the proteobacterium *Shewanella oneidensis* (HipA_So_)^30,31^. To investigate whether Lpg2370 is also autophosphorylated, we incubated purified recombinant Lpg2370 expressed in *E. coli* with the N6-benzyladenosine-5’-O-(3-thiotriphosphate) (N^6^-Bn-ATPγS) (Fig. 1c)^33^. Immunoblotting with anti-N^6^-Bn-ATPγS antibody detected a protein band corresponding to Lpg2370 (35 kDa) (Fig. 1d), which clearly indicated that Lpg2370 can be autophosphorylated. We next performed LC-MS/MS to identify the autophosphorylation site on Lpg2370. A mass shift of 79.97 Da (*m/z* = 684.85, *z* = 2) was consistently observed in the putative P-loop (53-MSVQGVQKK-61), revealing that the residue Ser54 within the P-loop is the autophosphorylation site (Fig. 1e). Taken together, these results suggest that Lpg2370 is a Ser/Thr kinase, though its substrates are currently unknown.

### Lpg2370 adopts a Ser/Thr kinase-like fold

To gain deeper insight into the molecular function of Lpg2370, we set out to determine its crystal structure. Diffraction phases for the SeMet-labeled Lpg2370 were determined using the single-wavelength anomalous diffraction method and the final structural mode was refined at 1.46 Å (Table 1).

**Table 1:** X-ray data collection and refinement statistics

Dataset	pHipT_Lp_	pHipT_Lp_–AMP–PNP	HipT_Lp_–HipS_Lp_
***Data collection***			
Wavelength (Å)	0.9792	0.9792	0.9792
Space group	P2_1_2_1_2_1_	P2_1_2_1_2_1_	P4_1_2_1_2
Cell dimensions			
a, b, c (Å)	52.95, 64.64, 90.42	39.81, 86.31, 91.88,	45.22, 45.22, 394.55
α, β, γ (°)	90.00, 90.00, 90.00	90.00, 90.00, 90.00	90.00, 90.00, 90.00
Resolution range (Å)	27.32–1.46 (1.51–1.46)	36.52–1.59 (1.65–1.59)	31.87–1.82 (1.885–1.82)
R_merge_	0.125 (1.08)	0.08 (1.22)	0.084 (1.35)
CC1/2	0.95 (0.87)	0.996 (0.870)	0.999 (0.872)
I/σ (I)	9.4 (2.00)	10.3 (2.20)	22 (2.60)
Completeness (%)	95.32 (90.51)	99.39 (98.12)	99.9 (99.70)
Multiplicity	8.57 (6.44)	12.3 (9.80)	24 (24.70)
***Refinement***			
Resolution (Å)	27.32–1.46 (1.51–1.46)	36.52–1.59 (1.65–1.59)	31.87–1.82 (1.885–1.82)
R_work_ (%)	17.53 (24.15)	17.20 (18.60)	18.59 (27.23)
R_free_ (%)	19.42 (25.06)	19.26 (21.40)	19.28 (21.32)
Ramachandran plot (%)			
Favored region	98.40	98.34	98.34
Allowed region	1.60	1.66	0.00
Outliers region	0.00	0.00	0.00

One crystal was used for the determination of each structure. Values in parentheses are for the highest-resolution shell.

**Fig. 2:**
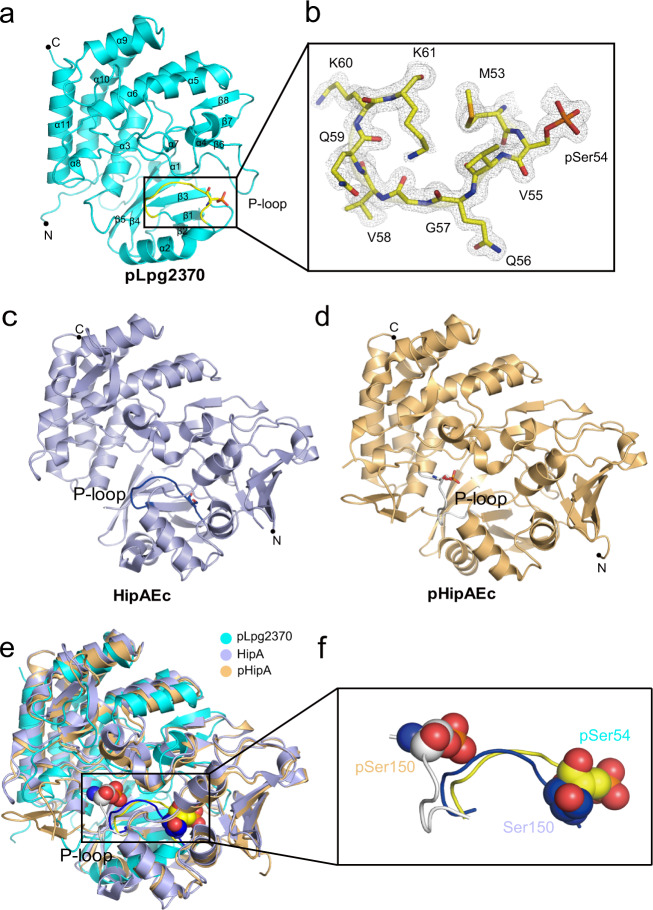
Crystal structure of pLpg2370. **a** Cartoon representation of pLpg2370. The N- and C-termini of pLpg2370 and its secondary elements of are labeled correspondingly. The P-loop is colored yellow and the pSer54 is shown in stick representation. **b** Detailed view of the P-loop with the pSer54. The 2Fo-Fc omit map is contoured at the 1.0 σ level and the P-loop residues are labeled. Crystal structures of the unphosphorylated HipA_Ec_ (PDB ID: 3TPB) (**c**) and pHipA_Ec_ (PDB ID: 3TPE) (**d**). The P-loops are labeled on both structures and colored blue and white, respectively. The P-loop serine/phosphoserine is shown as a stick representation. **e** Superimposition of pLpg2370 with the structures of pHipA and HipA. **f** Detailed view of the P-loops from the three superimposed structures. Ser150 in HipA_Ec_ and phosphoserines in pHipA_So_ and pLpg2370 are shown as spheres. Please note that the phosphorylated P-loop of Lpg2370 is bent toward N-lobe, similar to the unphosphorylated P-loop of HipA.

Like other members of the protein kinase superfamily, Lpg2370 has a globular kinase fold that can be further divided into N-lobe and C-lobe. The N-lobe, which contains the P-loop, is composed of sheets β1–5 sandwiched by helices α1 and α2, whereas the C-lobe is predominantly α-helical and consists of helix bundles α3–α6 and α8–α11 and a short β-sheet β6–8 (Fig. 2a). In line with the results of primary sequence analysis, there is no apparent structural similarity between Lpg2370 and the E3 ligase FANCL (Supplementary Fig. 2a), and residues 4–28 of Lpg2370, which are relatively well aligned with FANCL^25^, are a part of the typical kinase N-lobe. In agreement with our LC-MS/MS results, we can observe a phosphate group covalently attached to the Ser54 residue (Fig. 2b). However, unlike HipA_Ec_ and HipA_So_ whose P-loops are disordered upon serine autophosphorylation^29^, the electron density of the Lpg2370 P-loop is well defined in the present structure (Fig. 2b). Interestingly, the P-loop of Lpg2370 differs from the counterparts of typical protein kinases in that it contains a single glycine residue^34^. The positioning of the phosphorylated Ser54 (pSer54) is stabilized by the positively charged side chains of K40, R131, and R134, as well as hydrogen bonds between side chain of Gln56 and side chains of Asp145 and Lys201 (Supplementary Fig. 2b).

Dali search suggested that Lpg2370 shares the highest structural homology score with HipA_Ec_ and HipA_So_ (Supplementary Table 1)^35^. Lpg2370 and HipA_Ec_ superimpose with a relatively large root-mean-square deviation (RMSD) value of 3.846 Å over 221 Cα atoms (Fig. 2c, d). In addition to lacking a counterpart to the N-terminal region of HipA_Ec_ (i.e., helices α1–α4 and strands β1–β3 of HipA), Lpg2370 differs from HipA_Ec_ mainly in the configuration of the N-terminal lobe (Fig. 2c). One prominent difference is that the Lpg2370 P-loop with phosphorylated Ser54 is exposed to solvent in an orientation similar to that of unphosphorylated P-loop of HipA_Ec_, which upon serine autophosphorylation rotates by ~180° and bends away from the N-terminal lobe by 17.3 Å (Fig. 2e, f). Conversely, the six C-terminal α-helices of Lpg2370 and HipA_Ec_, including the catalytic residues and some ATP-binding residues, are almost perfectly aligned (Fig. 2e).

### Lpg2368–Lpg2369–Lpg2370 constitute the tripartite HipBST TA system of *L*. *pneumophila*

**Fig. 3:**
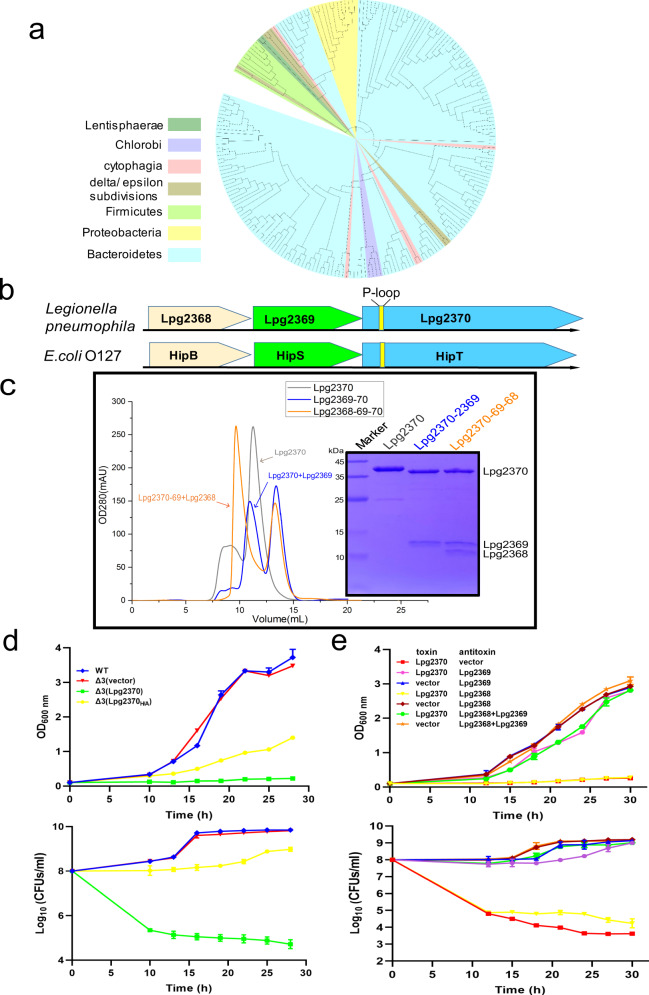
*L*. *pneumophila* genes *lpg2370*, *lpg2369*, and *lpg2368* belong to the same operon and constitute a tripartite HipBST family TA system. **a** The HipA-C-terminal domain-like proteins are found in bacteria, branches of the IQTree maximum likelihood phylogenetic tree of representative HipA-C-terminal sequences are colored by the taxonomic groupings as per the upper left panel. **b** Schematic of the *lpg2368–lpg2369*-*lpg2370* operon in *L. pneumophila* and the HipBST operon from *E. coli* O127:H6 strain. In the genome of *L. pneumophila*, *lpg2368, lpg2369*, and *lpg2370* (relative position of the conserved P-loop is highlighted in yellow) are located in the same operon and encode proteins that share homology with the HipBST TA system. **c** Comparison of elution profiles of Lpg2370 (11.3 mL), Lpg2370-Lpg2369 (10.9 mL), and Lpg2370-Lpg2369-Lpg2368 (9.6 mL) in size-exclusion chromatography (Superdex 75 increase column). The accompanying SDS-PAGE gel with peak fraction samples is provided on the right side. **d** Growth curve (upper panel) and the CFU count (bottom panel) of *L. pneumophila* ∆lpg2368-∆lpg2369-∆lpg2370 (∆3) expressing the kinase site active mutant H199A (H/A) and the wild-type Lpg2370, Lpg2370 causes cellular growth arrest upon induction, whereas the kinase site active mutant H199A (H/A) does not. **e** Growth curve (upper panel) and the CFU count (bottom panel) of overnight cultures *L. pneumophila* ∆lpg2368-∆lpg2369-∆lpg2370 (∆3) harboring pZL507 (pZL507:: *lpg2370*) or the empty pZL507 combined with pJL03 (pJL03:: *lpg2368*), pJL03 (pJL03:: *lpg2369*), pJL03 (pJL03:: *lpg2368–lpg2369*) or the empty low-copy-number pJL03 vector, as indicated, the tested bacterial strains were diluted in fresh AYE broth (supplemented with 10 μg/mL Gentamicin) to OD_600_ = 0.1 and split into 2-mL subcultures. 200 μM IPTG was added to induce the expression of Lpg2370, and 1% arabinose was added to induce the expression of Lpg2368, Lpg2369, or Lpg2368 + Lpg2369. In panels **d** and **e**, data shown are mean values ± SD (*n*  =  3 independent experiments).

Further analysis of Lpg2370 showed that proteins containing the C-terminal domain of HipA are widespread in bacteria (Fig. 3a). Moreover, the structural similarity between Lpg2370 and *E. coli* HipA and the fact that *E. coli* HipA along with HipB from the same genomic locus composes a type II TA system prompted us to examine the locus of *lpg2370*. Indeed, we found that *lpg2370* is preceded by open reading frames (ORFs) of *lpg2368* and *lpg2369*. Analogously to TA systems such as HipBA^28^, *lpg2368*, and *lpg2369* as well as *lpg2369* and *lpg2370* overlap by 4 bp. Further analysis suggested that *lpg2369* encodes a 102-aa protein similar to the N-terminal region of *E. coli* HipA and that *lpg2368* encodes a 72-aa protein homologous to the helix–turn–helix (HTH) domain of HipB (Fig. 3b and Supplementary Fig. 3a, b). Such locus organization is reminiscent of the HipBST TA module in *E*. *coli* O127:H6^17,18^, suggesting that the *lpg2368–lpg2369-lpg2370* locus is a potential tricistronic operon encoding component of a HipBST TA system.

In the HipBST TA system of *E*. *coli* O127:H6, the toxin HipT (denoted HipT_O127_) can form a heterotrimeric complex with the antitoxin HipS_O127_ and the HTH domain protein HipB_O127_^18^. We therefore performed size-exclusion chromatography, pull-down assays and isothermal titration calorimetry (ITC) to analyze interactions between Lpg2370, Lpg2369, and Lpg2368. The co-expressed 6×His-tag Lpg2369 and untagged Lpg2370 were co-eluted using Ni affinity chromatography, and the size-exclusion chromatography analysis revealed that the peak is shifted forward by 0.4 mL compared to the peak of Lpg2370 alone, suggesting Lpg2369 can interact with Lpg2370 (Fig. 3c). Moreover, size-exclusion chromatography indicated that Lpg2368 co-elutes with the co-expressed 6×His-tagged Lpg2369–Lpg2370 complex and binds to the Lpg2369–Lpg2370 complex assembled in vitro (Fig. 3c), which was then further confirmed by the pull-down assays (Supplementary Fig. 4). These results suggest that Lpg2370 directly interacts with Lpg2369, whereas Lpg2368 binds to a stable Lpg2369–Lpg2370 complex. Moreover, the results of ITC assays demonstrated that the dissociation constants between Lpg2370 and Lpg2369 and Lpg2370-Lpg2369 complex and Lpg2368 are 42 nM and 1.5 µM, respectively (Supplementary Fig. 5a, b), which is in agreement with the previously published data on HipBST_O127_^18^.

Given the established analogy between the *L*. *pneumophila* Lpg2368–Lpg2369–Lpg2370 operon and the HipBST TA system, we next aimed to functionally characterize Lpg2370 by investigating its potential toxicity to host bacteria. Heterogeneous expression of the recombinant Lpg2370 in *E*. *coli* had no observable effect on cell growth (Supplementary Fig. 6). The toxicity assays were performed in the *L. pneumophila* strain Lp02. To avoid undesirable effects from endogenous expression, we prepared deletion strain *∆lpg2368-∆lpg2369-∆lpg2370* (∆3) and examined bacterial growth upon overexpression of recombinant Lpg2370. Overexpression of Lpg2370 significantly inhibited the growth of *L. pneumophila*, both on plates and in liquid medium (Fig. 3d). Moreover, the catalytically inactive H199A (H/A) mutant failed to inhibit bacterial growth (Fig. 3d), indicating that the kinase activity of Lpg2370 is strictly required for its toxicity (Fig. 3d).

To assess the impacts of Lpg2368 and Lpg2369 on bacterial growth, we inserted *lpg2370* into the low-copy-number IPTG-inducible vector pZL507, and *lpg2368*, *lpg2369*, or *lpg2368–lpg2369* were separately inserted into the plasmid pJL03 with the arabinose-inducible pBAD promoter. Growth and viability of the *L. pneumophila* Δ3 strain carrying combinations of these plasmids was then monitored. Growth inhibition caused by the expression of Lpg2370 was counteracted by co-expression of Lpg2369, suggesting that Lpg2369 functions as the antitoxin (Fig. 3e). Co-expression of Lpg2368 and Lpg2369 was also found to counteract Lpg2370-dependent growth inhibition, whereas the expression of Lpg2368 without Lpg2369 could not prevent the growth inhibition (Fig. 3e). Taken together, these results are consistent with the findings on the *E. coli* O127:H6 HipBST module^17,18^ and demonstrate that Lpg2368, Lpg2369, and Lpg2370 from *L*. *pneumophila* constitute the tripartite HipBST TA system^18^ and will thus hereafter be referred to as HipT_Lp_, HipS_Lp_, and HipB_Lp_, respectively^17,18^.

### The kinase activity of HipT_Lp_ is likely independent of P-loop serine autophosphorylation

**Fig. 4:**
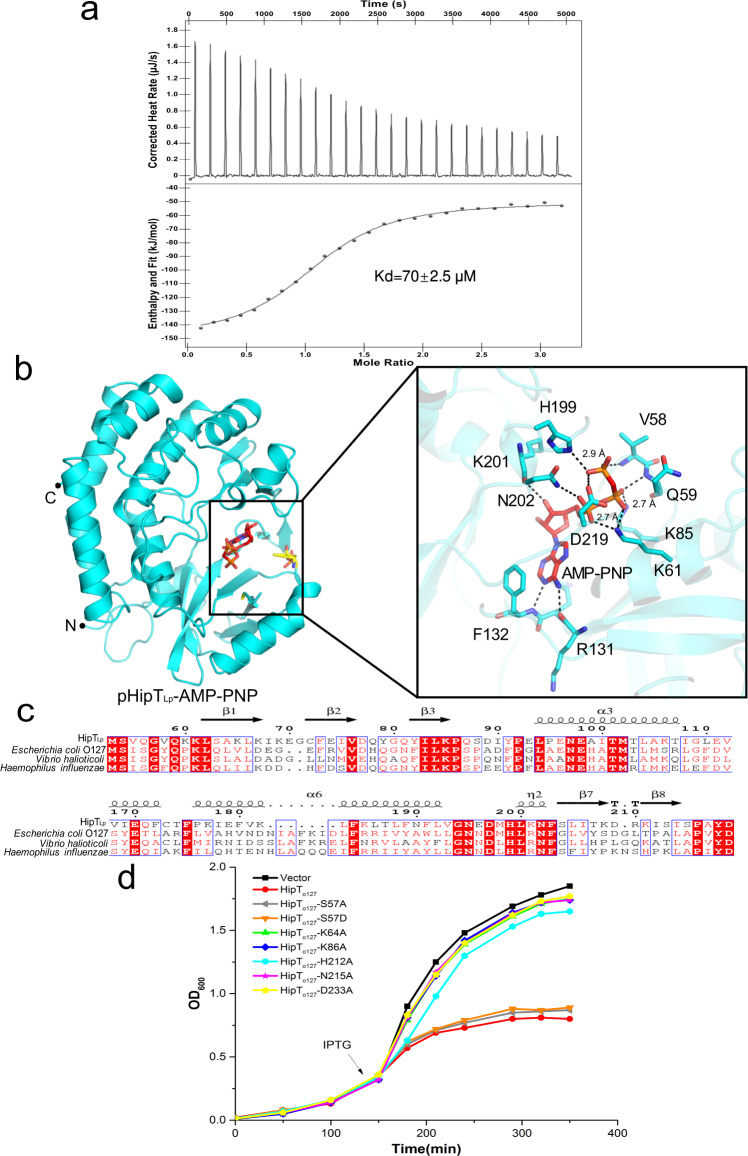
Crystal structure of the pHipT_Lp_–AMP–PNP complex. **a** Binding of AMP–PNP to pHipT_Lp_ monitored by ITC, the presented data is from a single ITC experiment. **b** Left: cartoon representation of the pHipT_Lp_–AMP–PNP complex. The N- and C-termini of pHipT_Lp_ are labeled. The bound AMP–PNP molecule and pSer54 are shown as sticks and colored red and yellow, respectively. Right: detailed view of the ATP-binding cavity of HipT_Lp_ and interactions formed with AMP–PNP. The distance between the interacting residues of HipT_Lp_ and AMP–PNP are in range of 2.7–3.3 Å, which was shown as sticks and hydrogen bonds are indicated with black dashed lines. **c** Sequence alignment of HipT variants from *L. pneumophila*, *Escherichia coli* O127:H6, *Vibrio halioticoli* and *Haemophilus influenzae* reveals conservation of the residues involved in ATP binding. The ATP-binding residues are encircled with black bold rectangles. **d** Growth curves of *E. coli* BL21(DE3) cells expressing recombinant wild-type HipT_O127_ or its mutant variants S57A, S57D, K64A, K86A, H212A, N215A, and D233A.

A comparison of the crystal structure of pHipT_Lp_ and the structures deposited in the PDB revealed that the autophosphorylated P-loop in HipT_Lp_ adopts an orientation similar to that of the P-loop in the crystal structure of *E. coli* HipA S150A mutant (Fig. 2f)^30^. This observation led us to speculate that pHipT_Lp_ can bind ATP. Thermal shift assays performed with the purified wild-type HipT_Lp_ revealed a 2.5 °C-increase in the melting temperature (Tm) in the presence of non-hydrolysable ATP analogue adenylyl-imidodiphosphate (AMP–PNP), suggesting that pHipT_Lp_ indeed binds ATP (Supplementary Fig. 7). Likewise, isothermal calorimetry determined that the dissociation constant between pHipT_Lp_ and AMP–PNP was about 70 µM (Fig. 4a), which is within the range of ATP-binding affinity expected for other kinases^29^.

To elucidate how pHipT_Lp_ binds ATP, we determined the crystal structure of pHipT_Lp_ in complex with AMP–PNP at 1.36 Å resolution (Table 1). The structure of pHipT_Lp_–ATP reveals that AMP–PNP is bound to the P-loop like in other representative kinases (Fig. 4b). The backbone of pHipT_Lp_ in the complex is virtually identical to the apo structure (RMSD = 0.35 Å), with the exception of P-loop that bends towards helix α2 to accommodate the AMP–PNP. ATP (AMP–PNP)-interacting residues appear to be conserved among the bacterial HipT toxins, implying a shared mechanism for ATP binding (Fig. 4c). In pHipT_Lp_, the γ-phosphate of AMP–PNP is stabilized by V58, H199, and D219, the β-phosphate forms hydrogen bonds with Q59, K61, and K85, whereas the α-phosphate interacts with K85 and N202. The adenosine moiety interacts with the main chain of K130 and forms π-stacking interactions with the side chain of F132 (Fig. 4b).

A previous study demonstrated that the kinase activity of *E. coli* HipA is essential for the growth arrest of host cells^36^, and cell growth was inhibited when HipT_O127_ was expressed in *E*. *coli* BL21 (DE3) cells^18^. To confirm the role of the residues involved into the ATP binding in the HipT toxins in vivo, HipT_O127_ TA is used to perform the growth inhibition assays due to the easy manipulation of *E. coli* compared to *L. pneumophila*. To investigate whether the above-mentioned residues responsible for ATP binding are essential for the kinase activity of HipT, we performed in vivo toxicity assays with HipT_O127_ variants in which residues corresponding to the S54 and the highly conserved ATP-binding residues of HipT_Lp_ were substituted with alanine. Intriguingly, mutation on the residues corresponding to S54 of HipT_Lp_ (S57A and S57D HipT_O127_) remain toxic to *E. coli* cells, whereas substitutions of K64 (K61), K86 (K85), H212 (H199), N215 (N202), and D233 (D219) of HipT_O127_ (corresponding residues in HipT_Lp_ are indicated in parentheses) eliminated the toxic phenotype (Fig. 4d). Taken together, these results suggest that unlike in *E. coli* HipA, HipT retains the ATP-binding ability independent of the autophosphorylation on the conserved S54 in the P-loop and that HipT uses a universal mode for ATP recognition.

### Structural basis for the toxin HipT_Lp_ recognition by the antitoxin HipS_Lp_

Although the toxic activity of HipT in the HipBST TA system has been demonstrated to be counteracted by the antitoxin HipS^17,18^, the underlying molecular mechanism remains unknown. We therefore sought to determine the structure of the HipT_Lp_–HipS_Lp_ complex. To express the HipT_Lp_–HipS_Lp_ complex, a ribosomal-binding site (RBS, AGGAGA)^37^ was introduced between the stop codon of HipS_Lp_ and the start codon of HipT_Lp_. The resultant HipS_Lp_–RBS-HipT_Lp_ was cloned into pET21a (+) vector. The crystal structure of the SeMet-labeled HipT_Lp_–HipS_Lp_ complex was determined and refined at 1.89 Å resolution (Table 1).

**Fig. 5:**
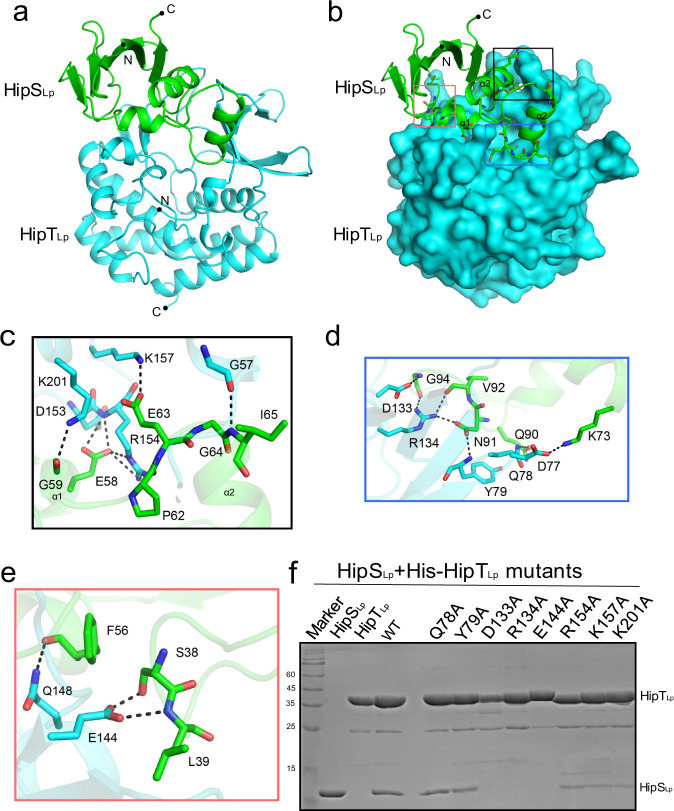
Structural basis for HipT_Lp_ recognition by HipS_Lp_. **a** Cartoon representation of the HipT_Lp_–HipS_Lp_ complex. HipT_Lp_ and HipS_Lp_ are colored cyan and green, respectively, and their N- and C-termini are labeled correspondingly. **b** Relative locations of three interacting regions formed between HipT_Lp_ and HipS_Lp_. The two proteins are color-coded as in panel A, and HipT_Lp_ is shown in surface representation. Interacting residues of HipS_Lp_ are shown as sticks and the three regions involved in interaction with HipS_Lp_ are encircled with black, blue, and red rectangles, respectively. **c**–**e** Detailed view of the three interacting regions formed between HipT_Lp_ and HipS_Lp_. Interacting residues are shown as sticks and hydrogen bonds are indicated with black dashed lines. **f** Pull-down assays performed with wild-type or mutant HipT_Lp_ carrying C-terminal 6×His-tag and untagged wild-type HipS_Lp_.

In the structure of HipT_Lp_–HipS_Lp_ complex, a copy of HipT_Lp_ and HipS_Lp_ each were observed per crystal asymmetric unit. Residues belonging to helices α1 and α2 of HipT_Lp_ were not visible in the electron density map, whereas the density of the remaining residues was unambiguous (Fig. 5a). All 102 residues of HipS_Lp_ were successfully built into the model, showing that HipS_Lp_ is a small single-domain protein composed of five β-strands and three α-helices. The overall structure of the HipT_Lp_-HipS_Lp_ complex is highly similar to *E. coli* HipA, with HipS_Lp_ and HipT_Lp_ aligning with the N- and C-terminal portions of *E. coli* HipA, respectively (Supplementary Fig. 8a). HipS_Lp_ superimposed with the N-terminus of *E. coli* HipA with an overall RMSD of 0.932 Å across 64 Cα. However, a notable difference can be observed on the β4–α2 loop of HipS_Lp_, which is twisted and rotated by ~45° with respect to its counterpart in the N-terminus of *E. coli* HipA (Supplementary Fig. 8b).

In the structure of HipT_Lp_–HipS_Lp_ complex, three α-helices of HipS_Lp_ form a helix bundle that sits above the cleft formed by the β-sheet in the N-terminal lobe of HipT_Lp_, whereas the β-strands form a flank region in HipS_Lp_ (Fig. 5b). HipS_Lp_ binds the toxin HipT_Lp_ via hydrogen bonding in three main interacting regions, which constitute more than 1100 Å^2^ of total buried surface area (Fig. 5c–e). The intermolecular interactions are mainly formed between helices α1, α2 and α1–α2 loop of HipS_Lp_ and helix α3 and strand β5 of HipT_Lp_. In the first interacting region, side chains of HipS_Lp_ E63 and HipT_Lp_ K157 form a salt bridge, side chain of HipS_Lp_ E58 engages in polar interactions with the main chain amide and side chain of HipT_Lp_ R154, and hydrogen bonds are additionally formed between main chain of HipS_Lp_ G59 and side chain of HipT_Lp_ K201 and main chains of HipS_Lp_ I65 and HipT_Lp_ G57 (Fig. 5c). The second interacting region includes a salt bridge between HipT_Lp_ D77 and HipS_Lp_ K73 and hydrogen bonds between (i) side chain of HipT_Lp_ D133 and HipT_Lp_ G94, (ii) side chain of HipT_Lp_ R154 and side chain of HipS_Lp_ N91 as well as main chain of HipS_Lp_ V92, (iii) side chain of HipS_Lp_ N91 and main chain of HipT_Lp_ Y79, and (iv) side chains of HipT_Lp_ Q78 and HipS_Lp_ Q90 (Fig. 5d). In the third interacting region, the side chain of HipT_Lp_ Q148 hydrogen bonds with the main chain of HipS_Lp_ F56, whereas the side chain of HipT_Lp_ E144 forms hydrogen bonds with the side chain of HipS_Lp_ S38 and the main chain of HipS_Lp_ L39 (Fig. 5e).

To verify the importance of these interactions for stable binding of HipS_Lp_ to HipT_Lp_, we performed pull-down assays with untagged wild-type HipS_Lp_ and wild-type or mutant HipT_Lp_ carrying a N-terminal 6×His-tag. The HipT_Lp_ mutants D133A, R134A, and E144A completely lost their ability to bind HipS_Lp_ and the mutants R154A, K157A, K201A exhibited severely reduced HipS_Lp_ binding, suggesting that these residues form key interactions with HipS_Lp_ (Fig. 5f).

### Molecular mechanism for toxin neutralization in the HipBST TA systems

**Fig. 6:**
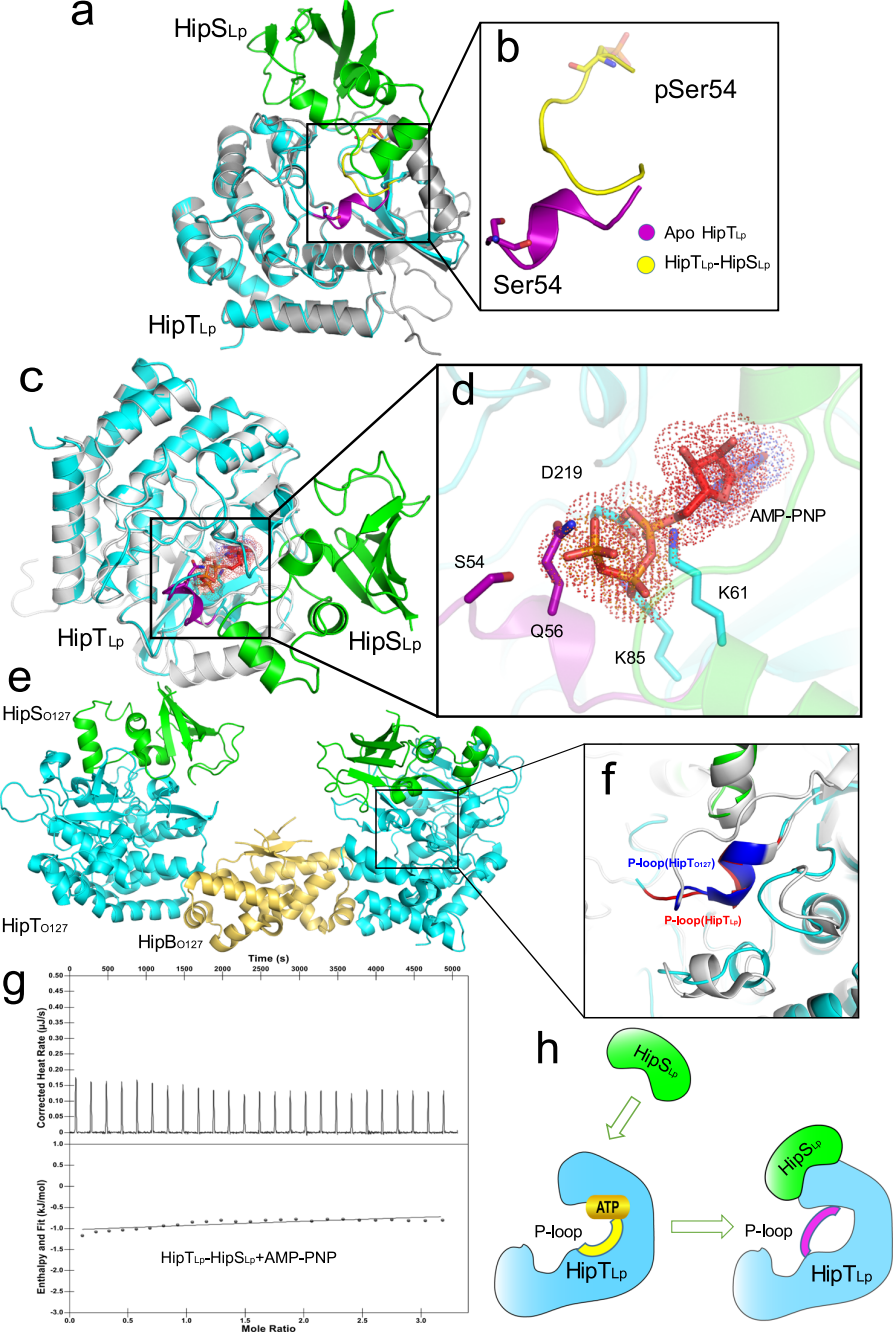
Toxin-neutralization mechanism in the HipBST TA systems. **a** Overlay of apo pHipT_Lp_ and structure of HipT_Lp_–HipS_Lp_ in cartoon representation. Apo pHipT_Lp_ is colored gray and its P-loop is colored yellow, whereas HipT_Lp_ from the binary complex is colored cyan and its P-loop is colored purple. HipS_Lp_ is colored green. **b** Close-up view of the overlay showing that HipS_Lp_ binding induces conformational change of the P-loop. **c** Overlay of the pHipT_Lp_–AMP–PNP and the structure of HipT_Lp_–HipS_Lp_ in cartoon representation. pHipT_Lp_ from the pHipT_Lp_–AMP–PNP is colored white. The HipT_Lp_–HipS_Lp_ complex is color-coded as in panel **a**. **d** Close-up view of the overlapping between HipT_Lp_ residues and AMP–PNP. AMP–PNP is shown as dotted surface mode and overlapping HipT_Lp_ residues are shown as sticks. **e** Crystal structure of HipBST_O127_ (PDB:7AB5) HipT_O127_, HipS_O127_ and HipB_O127_ were colored cyan, green and yellow orange, respectively. **f** Structural comparison between the HipT_Lp_–HipS_Lp_ and HipBST_O127._ The P-loops of HipT_Lp_ and HipT_O127_ are colored red and blue, respectively. **g** ITC thermogram and binding curve demonstrated that the HipT_Lp_–HipS_Lp_ complex does not display detectable affinity for AMP–PNP. **h** Proposed model for toxin-neutralization mechanism in the HipBST TA systems. The toxin HipT is a Ser/Thr kinase in which the P-loop motif is vital for ATP binding and subsequent substrate phosphorylation. Binding of the antitoxin HipS causes conformational changes in the P-loop, which blocks ATP binding and ultimately inhibits the kinase activity of HipT.

One of the most striking features of the HipBST TA systems is that the role of antitoxin is taken by HipS which corresponds to the N-terminal portion of HipA toxin from the *E. coli* HipBA system. To better understand how the toxic activity of HipT is neutralized by HipS, we reinspected and compared the structures of apo pHipT_Lp_, pHipT_Lp_–AMP–PNP complex, and HipT_Lp_–HipS_Lp_ complex. Apo pHipT_Lp_ and HipT_Lp_ from the HipT_Lp_–HipS_Lp_ complex superimpose with RMSD of 0.464 Å over 215 Cα atoms. Notably, Ser54 of HipT_Lp_ is phosphorylated in the structure of apo pHipT_Lp_ but not in the HipT_Lp_–HipS_Lp_ complex (please note that HipT_Lp_–HipS_Lp_ was co-expressed in *E. coli* BL21) (Fig. 2a, b and Supplementary Fig. 8c). Since the residue S54 is phosphorylated when HipT_Lp_ is expressed alone, we wondered whether the phosphorylation on S54 influences the interaction between HipT_Lp_-HipS_Lp_ and HipB_Lp_. Size-exclusion chromatography revealed that the phosphorylation state of S54 does not appear to have a noticeable effect on interactions between HipT_Lp_-HipS_Lp_ and HipB_Lp_ (Supplementary Fig. 9). Moreover, structural comparison suggests that the P-loop of HipT_Lp_, which encircles ATP and is critical for catalytic activities in typical Ser/Thr kinases, underwent a conformational change from loop to helix upon HipS_Lp_ binding (Fig. 6a, b). Such allosteric regulation induced by the antitoxin binding has not been observed in *E. coli* HipBA TA system^29^. A conformational change similar to the loop-to-helix change of the HipT_Lp_ P-loop in HipT_Lp_-HipS_Lp_ can also be observed in the recently released structure of HipBST_O127_ trimer^19^ (Supplementary Fig. 10), suggesting a common mechanism of toxin neutralization.

These observations led us to hypothesize that the loop-to-helix conformational transition induced upon HipS_Lp_ binding may obstruct the access of ATP to the kinase active site, resulting in inhibition of the HipT_Lp_ kinase activity. Superimposition of the structures of pHipT_Lp_, pHipT_Lp_–AMP–PNP, and the HipT_Lp_–HipS_Lp_ further revealed that P-loop in the HipT_Lp_–HipS_Lp_ complex overlaps with AMP–PNP in the pHipT_Lp_–AMP–PNP complex (Fig. 6c, d and Supplementary Fig. 11). More specifically, the γ-phosphate and β-phosphate groups of AMP–PNP would clash with the side chains of Q59 and D219, respectively, whereas the α-phosphate would clash with the side chains of K61 and K85 (Fig. 6d). This may account for the unphosphorylated state of the P-loop in the HipT_Lp_–HipS_Lp_ complex when they were co-expression (Supplementary Fig. 8c). Such conformational change also occurs in the P-loop of HipBST_O127_, suggesting that a similar mechanism is utilized by HipBST_O127_^19^ (Fig. 6e, f). To further verify whether ATP binding is abolished, we measured the binding affinity between the HipT_Lp_–HipS_Lp_ complex and AMP–PNP with ITC and found that HipT_Lp_ completely lost the AMP–PNP binding affinity when binding with HipS_Lp_ (Fig. 6g). Consistent with these results, the thermal stability of the HipT_Lp_-HipS_Lp_ complex did not change upon the addition of 4 mM AMP–PNP (Supplementary Fig. 11a). Considering that HipA_Ec_ in autophosphorylated form can bind ADP and AMP but not ATP^30^, we also investigated whether the HipT_Lp_–HipS_Lp_ complex binds ADP and AMP. Again, the results of ITC experiments suggested that the HipT_Lp_–HipS_Lp_ complex has no detectable affinity for ADP or AMP, even at concentrations of 1 mM (Supplementary Fig. 12). The allosteric regulation of the P-loop induced by the antitoxin binding was also observed in the HipBST_O127_^19^. Together, these findings suggest that HipS_Lp_ binding induces conformational changes in the P-loop of HipT_Lp_, which blocks ATP binding and consequently inhibits the HipT_Lp_ kinase activity (Fig. 6h).

## Discussion

Although the biological functions of TA systems are often ambiguous and debatable, recent advances in the field and the discovery of numerous novel TA modules increasingly support their roles in viral defense or plasmid stability^4,7^ and interactions between hosts and their mobile genetic elements. Among known TA modules, the type II TA systems are most well understood. Type II TA systems typically consist of two components, though several tripartite type II TA modules have been identified, such as the *Rv1955-Rv1956-Rv1957* TA-chaperone (TAC) system of *Mycobacterium tuberculosis*^38,39^, and the recently discovered *E*. *coli* HipA-like TA system HipBST of *E*. *coli* O127: H6^18^.

Most type II antitoxins are composed of an N-terminal DNA-binding domain that regulates transcription from the TA locus through direct interaction with the promoter, and a C-terminal region responsible for the toxin binding and inhibition^40^. HipBA modules are representative type II TA systems and are ubiquitous in bacteria^28^. Based on the similarity searching using *E. coli* HipA against the sequenced microbial genomes, a three-component widespread HipBST TA was found and experimentally verified^18^. Later bioinformatic analysis has suggested the presence of several other HipBA-like TA in numerous bacterial species^17^. While the toxin HipT_O127_ of HipBST_O127_ was found to exert its toxic function by phosphorylating TrpS, and in spite of the similarity between HipB_O127_ and *E. coli* HipB, HipB_O127_ however cannot neutralize the HipT_O127_ kinase. This task was taken over by HipS, a small protein with homology to the N-terminal part of *E. coli* HipA^18,19^. Our current study also experimentally identifies a tripartite HipBST_Lp_ TA in which the antitoxin HipS_Lp_ instead of HipB_Lp_ restores the growth inhibition induced by HipTLp in human pathogen *L. pneumophila*, which is in agreement with the HipBST_O127_ TA^18^.

Together with the preprint study in ref. 19, our study identifies and elucidates the toxin-neutralization mechanism in the tripartite HipBST TA systems of *L. pneumophila*. It is clear that the toxin neutralization mechanism in HipBST systems is notably different from the corresponding mechanism in HipBA TA system of *E*. *coli*^18^. The structural study by Bærentsen et al. on the *E. coli* O127:H6 HipBST found that HipT adopts an inactive conformation in the HipBST complex that prevents ATP binding^19^. However, it was unclear whether the blockage of ATP binding arises from the binding of the antitoxin HipS or autophosphorylation of the conserved P-loop serine^19^. Our structure of the HipT_Lp_–HipS_Lp_ complex clearly shows the toxicity of HipT_Lp_ is neutralized directly upon HipS_Lp_ binding, leading to blocking of the ATP-binding site through steric hindrance. Although this study demonstrates that HipB_Lp_ forms a heterotrimer with the HipT_Lp_–HipS_Lp_ complex, we failed to obtain crystals of the heterotrimer structure after extensive crystal screening. However, the available structure of *E. coli* HipBST shows that HipB binds to HipT but does not interact with the kinase active site^19^. In addition, overall architecture of HipBST_O127_ system is reminiscent of the structure of HipBA_So_ system in the HipBA_So_-DNA complex, implying that HipBST can also bind DNA^19^. Induction of HipB_O127_ was found to be required and sufficient for the transcriptional repression of the HipBST TA^19^. Thus, it seems that the toxin neutralization and the autoregulation of the HipBST TA are carried out by the antitoxin HipS and HipB, respectively^19^. Nevertheless, the exact role of HipB in the HipBST systems remains to be determined.

To the best of our knowledge, HipT_Lp_ is the only identified toxin of TA systems secreted into host cells via the type IV secretion system Dot/Icm. Nevertheless, the employment of TA toxins by the pathogenic bacteria during host cell infection is not unprecedented. For instance, the type III secretion system effector AvrRxo1-ORF1 from *Xanthomonas oryzae* pv*. oryzicola* constitutes a type II TA system with adjacent AvrRxo1-ORF2^41^. AvrRxo1 can phosphorylate NAD in planta, leading to the suppression of the flg22-triggered ROS burst^42^. Moreover, previous investigation of fourteen effectors from *Burkholderia gladioli* revealed that the restriction endonuclease Tox-Rease-5 domain-containing effector TaseT_Bg_ can be employed to inhibit the growth of co-habiting bacterial species, and its activity is counteracted by the associated immunity protein TsiTBg^43^. Apart from the effectors LegK1–K5 and LegK7, HipT_Lp_ is the sixth effector known to function as a Ser/Thr kinase in *L. pneumophila*. LegK1 phosphorylates NF-κB to activate the genes expression involved in inflammation during infection^44^. Phosphorylation of Hsp70 at T495 by LegK4^45^, leading to the inhibition of protein synthesis. LegK7 phosphorylates a conserved scaffold protein MoB1, hijacking the hippo pathway to promote its survival^33,46^. The exact substrate of HipT_Lp_ in its host remains to be identified in future studies. In summary, these results suggest that TA systems could serve as a reservoir for additional secreted effectors, which sheds light on the evolutionary links between the TA system and the effectors secreted by the pathogenic microorganisms.

## Methods

### Bacterial strains and growth conditions

The *L. pneumophila* strain Philadelphia-1 derivative Lp02^47^ was used as the progenitor of all derivative strains used in this study. To construct *∆lpg2368*-*∆lpg2369-∆lpg2370* triple-deletion mutant strain of *L*. *pneumophila* (termed ∆3), we first constructed deletion plasmid by cloning the upstream and downstream flanking regions into pSR47S. Briefly, the 1.2-kb fragment located upstream of *lpg2368* and the 1.2-kb fragment located downstream of *lpg2370* were obtained by PCR using ∆3 A1/∆3 A2 and ∆3 B1/∆3 B2 primer pairs and high-fidelity FastPfu DNA polymerase (TransGen) (Supplementary Table 2), respectively. The amplified PCR products were used as templates to produce a DNA fragment containing the flanking regions by fusion PCR with primers ∆3 A1/∆3 B2 and high-fidelity FastPfu DNA polymerase (TransGen). After digestion with BamHI and SalI, the DNA fragment was inserted into BamHI/SalI-digested pSR47S^48^. The deletion plasmid was then introduced into Lp02 by triparental mating, and conjugants were selected on CYET plates containing kanamycin (20 μg/mL) and streptomycin (50 μg/mL). Deletion mutants were verified by standard colony PCR techniques using primers ∆3 A1/∆3 B2 and 2×Taq master mix (Novoprotein) from colonies grown on CYET plates containing 5% sucrose^49^. Moreover, the *lpg2370*, *lpg2368*, *lpg2369*, and *lpg2368–lpg2369* genes were inserted into pZL507 or pJL03 as BamHI/SalI fragments to construct pZL507-Lpg2370, pJL03-Lpg2368, pJL03-Lpg2369, and pJL03-Lpg2368-Lpg2369 plasmids, which were then introduced into the *∆lpg2368∆lpg2369∆lpg2370* strain. For effector translocation, fresh single colonies of Lp02 or Lp03 harboring expression plasmid for TEM-1-Lpg2370, TEM-1-RalF, or TEM-1-FabI fusion proteins were streaked onto BCYE plates 2 days before infection.

### TEM-1 β-lactamase translocation assays

To test Dot/Icm-dependent transfer of the fusion proteins into host cells, *L. pneumophila* cells expressing the fusion proteins were grown in the presence of 0.5 mM IPTG to post-exponential phase and used to infect monolayers of RAW264.7 cells that were seeded in 96-well plates at an MOI of 20. The CCF4-AM substrates (Invitrogen, Carlsbad, CA) were mixed with medium in the wells two hours after infection. After further incubation for 1 hour at room temperature, infected cells were inspected under a Nikon IX-80 fluorescence microscope equipped with a β-lactamase FL-Cube (U-N41031, Chroma Technology Corp, Bellows Falls, VT). Images of infected cells were obtained using a DP-72 color fluorescence camera (Olympus). Translocation of the β-lactamase chimeras was assessed by the presence of cells emitting blue fluorescence signals. The percentage of infected cells was determined by counting the number of cells emitting blue fluorescence in specified areas of the wells. TEM-1-RalF and TEM-1-FabI fusion proteins were used as positive and negative controls, respectively. Experiments were performed in triplicate, and at least 300 cells were counted in each sample.

### Protein expression

DNA fragments encoding full-length HipT_Lp_, HipS_Lp_, and HipB_Lp_ or their variants were inserted into pET21a (+) containing a N-terminal 6×His-tag, respectively. To obtain HipT_Lp_–HipS_Lp_ complex, HipS_Lp_–HipT_Lp_ was cloned in the pET21a (+) following previously described methods^37,50^ to express the 6×His HipT_Lp_–HipS_Lp_. The recombinant plasmids were transformed into *E. coli* BL21 (DE3) cells for protein expression. Overexpression was induced in log phase cultures (OD_600_ ≈ 0.6) by cooling cultures on ice for 20 min and adding IPTG to a final concentration of 0.4 mM, followed by overnight incubation at 16 °C and 220 rpm. The overexpression of the wild-type HipT_Lp_ did not lead to the growth arrest in *E. coli*. After target protein expression, cells were pelleted by centrifugation and resuspended in buffer A (50 mM Tris-HCl, pH 8.0, 100 mM NaCl). The cells were then lysed by ultrasonication and the lysate was centrifugated at 17,000×*g* and 4 °C for 30 min. The supernatant was loaded onto Ni^2+^-NTA column (Qiagen) for purification of target recombinant proteins. After washing with 100 mL of buffer A supplemented with 50 mM imidazole, the target proteins were eluted with buffer A supplemented with 250 mM imidazole. Fractions containing the target protein were pooled, concentrated to 0.5 mL and then purified with Superdex 75 increase column (GE Healthcare) equilibrated with buffer B (20 mM Tris-HCl, pH 8.0, 150 mM NaCl).

Selenomethionine (SeMet)-labeled HipT_Lp_ and HipT_Lp_–HipS_Lp_ were expressed in M9 medium supplemented with 2 mM MgSO_4_, 0.1 mM CaCl_2_, 0.5% w/v glucose, 2 mg/L biotin, 2 mg/L thiamine, and 0.03 mg/L FeSO_4_. When bacterial cultures reached OD_600_ of 1.0, final concentrations of 100 mg/mL of phenylalanine, lysine, and threonine, and 50 mg/mL of isoleucine, leucine, valine, and of SeMet (Chemie Brunschwig) were added in form of solid powder, after which the cultures were incubated for 30 min. Protein expression was then induced with 0.2 mM IPTG, and the cultures were further incubated on a shaker at 16 °C for 20 h. Cells were collected at 5000×*g* for 15 min and 4 °C and resuspended in the lysis buffer (50 mM Tris pH 8.0, 100 mM NaCl, 5 mM β-mercaptoethanol). Protein purification was performed as described above.

### In vitro kinase assays

Purified Lpg2370 (1 µg) was resolved by SDS-PAGE and transferred to the PVDF membrane using a Bio-Rad wet transfer system. The membrane was blocked with 5% milk for 1 h at room temperature. The membrane was incubated overnight at 4 °C with a primary antibody against thiophosphate ester (rabbit anti-thiophosphate ester (ab92570), Abcam, dilution 1:4000 v/v). The membrane was then washed three times with TBST before being incubated with a secondary antibody (HRP-conjugated AffiniPure goat anti-rabbit (Cat No. SA00001-2), Proteintech, dilution 1:5000 v/v) for 1 h at room temperature. The membrane was washed three times with TBST, and proteins were detected using an ECL detection reagent.

### Liquid chromatography-mass spectrometry (LC-MS) analysis

LC-MS was used to analyze autophosphorylation of purified recombinant Lpg2370. After staining of gels with Coomassie blue, excised gel segments were subjected to in-gel trypsin digestion and dried. Electrospray ionization mass spectrometry (ESI-MS) was performed using an integrated HPLC/ESI-MS system (1260 Infinity, Agilent Technologies/amaZon SL, Bruker Corporation) equipped with a Luna 5 µm C18 column (100 Å, 250 × 4.60 mm, 5 µm). Peptides were dissolved in 10 µl 0.1% formic acid and were auto-sampled directly onto a homemade C18 column (35 cm × 75 μm i.d., 1.9 μm 100 Å). Samples were then eluted for 60 mins with linear gradients of 3–35% acetonitrile in 0.1% formic acid at a flow rate of 300 nl/min. The mass spectrometer was equipped with a CaptiveSpray source. Survey scans were recorded over 100–1700 *m/z* range and the mass spectra data were acquired with a timsTOF Pro mass spectrometer (Brucker) operated in PASEF mode. PASEF setting: 10 MS/MS scans (total cycle time 1.27 s), charge range 0–5, active exclusion for 0.4 min, Scheduling Target intensity 10000, Intensity threshold 2500, CID collision energy 42 eV.

The raw files generated from LC-MS/MS were analyzed by Peaks Studio X software (Bioinformatics Solutions Inc., Waterloo, ON, Canada) against the input of the Lpg2370 amino acid sequence. The following database search criteria were set to: enzyme, trypsin; variable modification, phosphorylation, precursor ion mass tolerance, 10 ppm; MS/MS fragment mass tolerance, 0.02 Da; tryptic enzyme specificity with two missed cleavages allowed. Identifications were filtered according to mass accuracy and 1% false discovery rate.

### Protein crystallization and collection of crystallographic data

The purified target protein was concentrated to 0.5 mL and loaded onto a Superdex 75 increase column (GE Healthcare) Fractions containing purified proteins were then concentrated at 4000×*g*, 4 °C to ~15 mg/mL using an Amicon Ultra 30 K centrifugal filter. To obtain HipT_Lp_–AMP–PNP complex, the purified HipT_Lp_ was incubated with AMP–PNP at a 1:1.2 molar ratio at 4 °C for 30 min and concentrated to about 12 mg/mL.

For crystallization of SeMet-labeled HipT_Lp_, HipT_Lp_–AMP–PNP complex, and SeMet-labeled HipT_Lp_–HipS_Lp_ complex, the purified and concentrated protein samples were mixed with the reservoir solution at equal volumes and crystallized using the sitting drop vapor diffusion method at 16 °C. Initial crystals of SeMet-labeled HipT_Lp_ and HipT_Lp_–AMP–PNP complex were obtained within three days in condition containing 8% Tacsimate (pH 6.0) and 20% w/v PEG 3350. Initial crystals of the SeMet-labeled HipT_Lp_-HipS_Lp_ complex were obtained in the condition containing 0.1 M sodium acetate (pH 7.0) and 12% v/w PEG 3350. After extensive optimization, diffraction-quality crystals of SeMet-labeled HipT_Lp_ and the HipT_Lp_–AMP–PNP complex were grown in the presence of 10% Tacsimate (pH 6.2) and 20% v/w PEG 3350. Diffraction-quality crystals of the HipT_Lp_–HipS_Lp_ complex were grown in the presence of 0.1 M sodium acetate (pH 7.2) and 15% w/v PEG 3350. Harvested crystals were preserved in the respective reservoir solutions supplemented with cryoprotectant and flash-frozen in liquid nitrogen.

### Structure determination and refinement

All X-ray diffraction data were collected at the BL-02U1 station of the Shanghai Synchrotron Radiation Facility (SSRF). Single-wavelength anomalous diffraction (SAD) datasets of SeMet-labeled HipT_Lp_ and SeMet-labeled HipT_Lp_–HipS_Lp_ complex were obtained at high resolution and the data were processed with the HKL-2000 package^51^. Autosol program of PHENIX package was used for SAD phasing and initial model building. Residues 72–312 of HipT_Lp_ were auto-built, whereas the residues 1–71 residues were built by iterative manual building in Coot^52^. Structure refinement was carried out with PHENIX^53^. The HipT_Lp_–AMP–PNP binary complex was determined using the molecular replacement method with the structure of HipT_Lp_ as the search model. Structure quality was analyzed during PHENIX refinements and later validated in the PDB validation server. Detailed crystallographic and structure refinement data are listed in Table 1. Structural images were generated using PyMol (Schrödinger, LLC).

### Mutagenesis

Base substitutions in this study were introduced using two pairs of complementary primers (sense and antisense strand primers) containing the desired mutation. The primers used in this study were listed in Supplementary Table 2. All constructs were verified by DNA sequencing.

### Isothermal titration calorimetry (ITC)

ITC experiments were performed in Nano ITC Low Volume (TA instruments). All samples were prepared in the buffer containing 20 mM HEPES (pH 8.0) and 150 mM NaCl. Typically, the titrant concentration in the syringe was 200–500 μM, and the titrand concentration in the reaction cell was 10–20 μM. Titration was conducted at 25 °C using multiple injection method with 150 s intervals. Obtained data were integrated, corrected, and analyzed using the NanoAnalyze software (TA Instruments) with a single-site binding model.

### Pull-down assays

To perform pull-down assays, wild-type or mutant HipT_Lp_ carrying C-terminal 6×His-tag were incubated with Ni-agarose beads for 30 min and then washed twice with buffer containing 20 mM Tris-HCl (pH 8.0) and 150 mM NaCl. The beads were then incubated with untagged wild-type HipS_Lp_ (6×His-tag was previously cleaved with TEV protease) for 1 h and then washed twice. The proteins were eluted from beads using a buffer containing 20 mM Tris-HCl (pH 8.0), 150 mM NaCl, and 250 mM imidazole. Eluted samples were analyzed using SDS-PAGE analysis.

### In vivo toxicity assays

For in vivo toxicity assays of HipT from *E. coli* serotype O127:H6 (denoted HipT_O127_) to *E. coli*, which was performed by expressing the *hipT*_*O127*_ gene or its mutant variants in *E*. *coli* BL21(DE3). The gene encoding HipT_O127_ was synthesized and cloned into pET21a (+) vector. Plasmids encoding HipT_O127_ single-point mutants S57A, S57D, K64A, K86A, H212A, N215A, and D233A were prepared using site-specific mutagenesis, and the primers are listed in Supplementary Table 2. Transformed *E. coli* BL21 (DE3) cells were plated on agar and a single bacterial colony was transferred to 10 mL LB medium for culturing. Expression of wild-type HipT_O127_ and its mutant variants was induced with IPTG at 0.2 mM concertation when the bacterial cultures reached OD_600_ = 0.5, after which the bacterial growth curve was measured every 30 min for 6 h.

For the Lpg2370 in vivo toxicity assays in *L. pneumophila*, Lpg2370, Lpg2370-Lpg2369, Lpg2370-Lpg2368_,_ Lpg2370-Lpg2369-Lpg2368 were overexpressed in *L. pneumophila* Lp02 or the ∆*lpg2368*∆*lpg2369*∆*lpg2370* deletion strain. Overnight cultures of the tested bacterial strains were diluted in fresh AYE broth to OD_600_ = 0.1 and split into 2-mL subcultures into which different concentrations of arabinose or IPTG were added. The subcultures were then grown at 37 °C with constant rotation at 180 rpm. Cell viability was assessed by readout of OD_600_ value every 3 h and plotting the values on a log scale.

### Thermal shift assays

Thermal shift assays were performed using 1 mg/mL phosphorylated HipT_Lp_ or its mutants incubated with ATP/AMP–PNP and varied concentrations of nucleotides (0–4 mM) in 50 mM Tris-HCl (pH 8.0) and 150 mM NaCl. Then the mixture was loaded in 96-well PCR plates, the fluorescence signals were recorded as a function of temperature using Prometheus NT.48 (NanoTemper Technologies) in FRET mode. Fluorescence intensity was measured at Ex/Em of 350/330 nm. The temperature gradient range was set as 20–95 °C with a 0.5 °C ramp over the course of 30 s. Control assays were conducted in the same buffer without ATP/AMP–PNP. The thermal unfolding value (Tm) for pHipT_Lp_ was calculated using the curve fitting software PR.ThermControl (NanoTemper Technologies).

### Phylogenetic analysis

The sequence of Lpg2370 was blasted in the Uniprot (https://www.uniprot.org/) and all the hit sequences were used for sequence alignment in ClustalW, which was used to build the phylogenetic tree using MEGA software^54^. The phylogenetic tree was visualized using iTOL^55^.

### Statistics and reproducibility

The TEM-1 β-lactamase translocation assays experiments in Fig. 1a, the western blotting experiment in Fig. 1d, the pull-down assays in Figs. 3c, 5f, Supplementary Fig. 4, and Supplementary Fig. 9 were performed at least triplicate at two independent times.

## Supplementary information

Supplementary Information

Peer Review File

reporting-summary

## Data Availability

The atomic coordinates and structure factors of the autophosphorylated toxin HipT_Lp_, the complex of HipT_Lp_ with AMP–PNP, and HipT_Lp_-HipS_Lp_ binary complex have been deposited in the Protein Data Bank under the accession codes 7VKC, 7WCF, and 7VKB.  Source data are provided with this paper.

## Source data

Source data
