# Supplementary material for: Molecular mechanism of toxin neutralization in the HipBST toxin-antitoxin system of Legionella pneumophila
Source: Nat Commun. 2022 Jul 26;13:4333. doi: 10.1038/s41467-022-32049-x (PMC9325769; doi:10.1038/s41467-022-32049-x)
Supplement: Supplementary file 1 — Supplementary Information [file 41467_2022_32049_MOESM1_ESM.pdf]

Supplementary Information

Molecular mechanism of toxin neutralization in the HipBST toxin-antitoxin system of *Legionella pneumophila*

Xiangkai Zhen[1#], Yongyu Wu[1#], Jinli Ge[2#], Jiaqi Fu[3], Le Ye[1,] Niannian Lin[1], Zhijie Huang[1], Zihe Liu[1], Zhao-qing Luo[3], Jiazhang Qiu[2*], Songying Ouyang[1,4*]

Xiangkai Zhen[1#], Yongyu Wu[1#], Jinli Ge[2#], Jiaqi Fu[3], Le Ye[1,] Niannian Lin[1], Zhijie Huang[1], Zihe Liu[1], Zhao-qing Luo[3], Jiazhang Qiu[2*], Songying Ouyang[1*]

1. Provincial University Key Laboratory of Cellular Stress Response and Metabolic Regulation, the Key Laboratory of Innate Immune Biology of Fujian Province, Biomedical Research Center of South China, Fujian Normal University, Fuzhou, 350117, China.

2. State Key Laboratory for Zoonotic Diseases, College of Veterinary Medicine, Jilin University, Changchun, China.

3. Purdue Institute for Inflammation, Immunology and Infectious Disease and Department of Biological Sciences, Purdue University, West Lafayette, IN, USA.

4. Key Laboratory of OptoElectronic Science and Technology for Medicine of the Ministry of Education, College of Life Sciences, Fujian Normal University, Fuzhou, 350117, China.

[#]These authors contributed equally to this study.

*Correspondence: Songying Ouyang (ouyangsy@fjnu.edu.cn) or Jiazhang Qiu (qiujz@jlu.edu.cn)

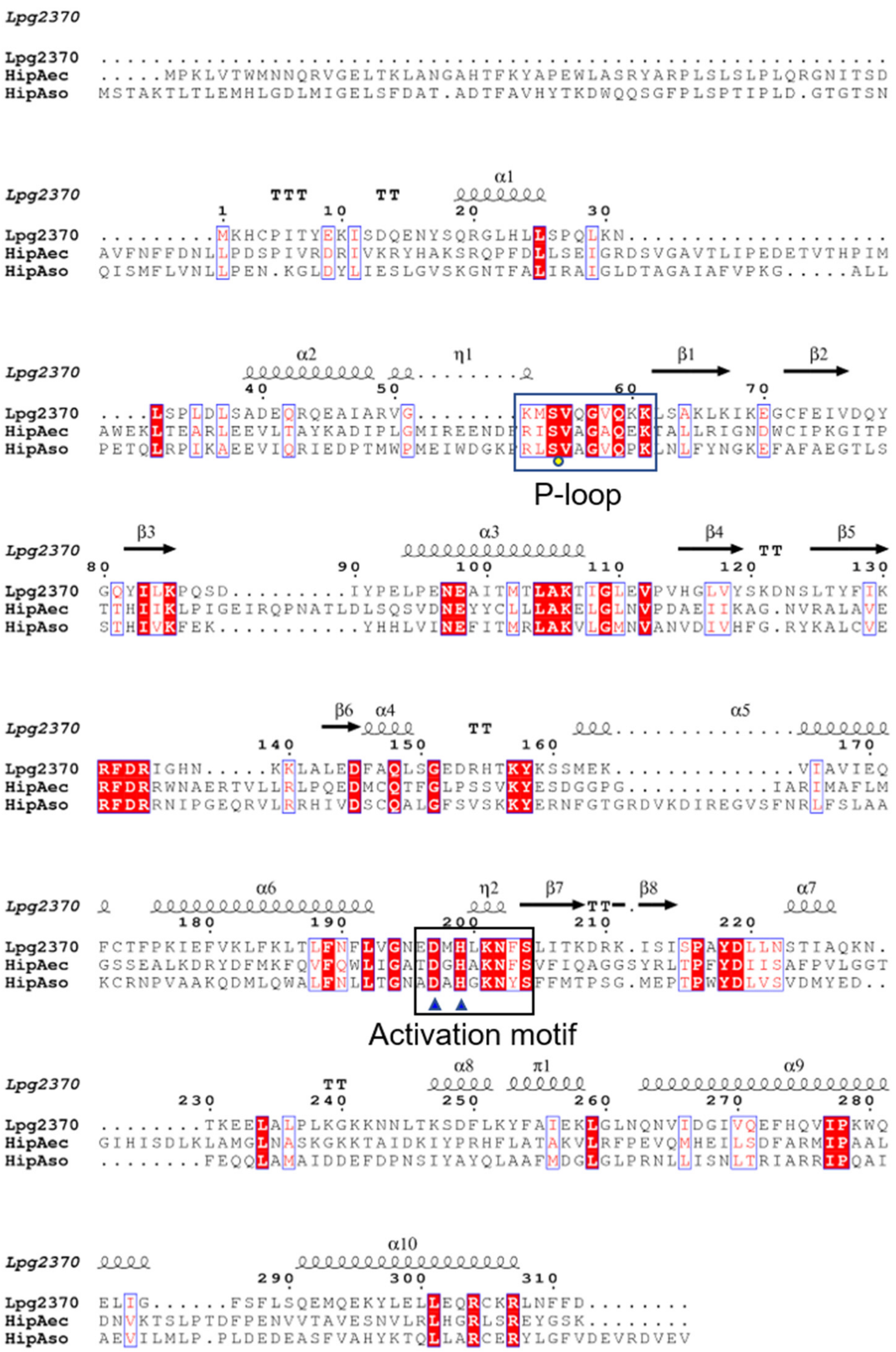

Supplementary Fig.1. Multiple sequence alignment of Lpg2370 with the Ser/Thr kinase toxin HipA from *E. coli* K-12 and *Shewanella onesidensis* suggests that Lpg2370 has a conserved signature P-loop with RISVAGAQ motif characteristic of Ser/Thr kinases. The conserved P-loop and the catalytic loop were boxed and the secondary structure elements of Lpg2370 as observed in the determined structure are indicated above the sequences. The highly conserved residues are highlighted in red. The phosphorylated serine in the P-loop (S54)

is indicated by a green circle under the aligned sequences. The key residues in the activation motif (D197 and H199) that are essential for the kinase activity are indicated by blue triangles under the aligned sequences.

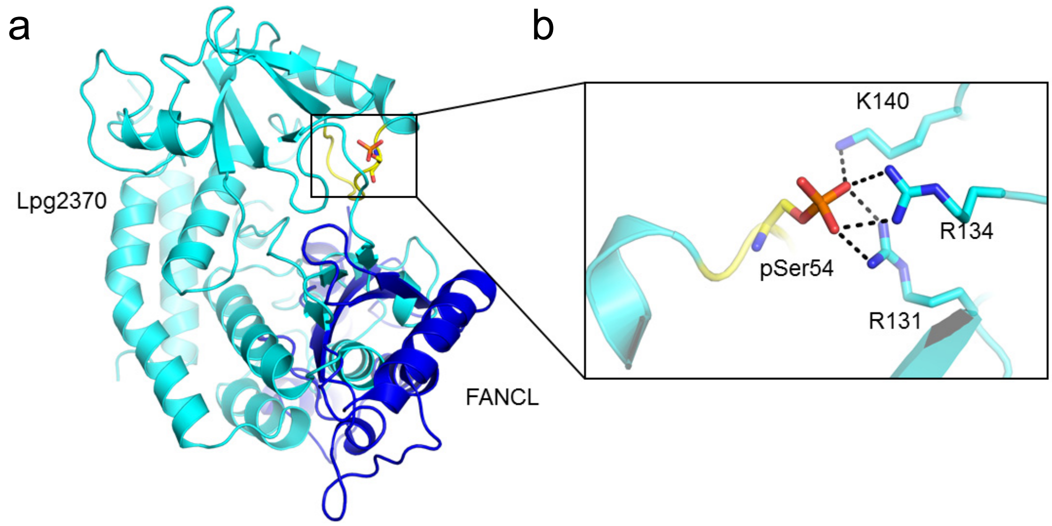

Supplementary Fig.2. Structural alignment of Lpg2370 and the E3 ligase FANCL (PDB ID: 4CCG). (**a**) Superimposition of Lpg2370 (cyan) and FANCL (blue) structures reveals no similarities between the two proteins. (**b**) Close-up view of the P-loop of Lpg2370. The P-loop is colored yellow. The pSer54 and side chains of residues K140, R131, and R134 stabilizing are shown as sticks.

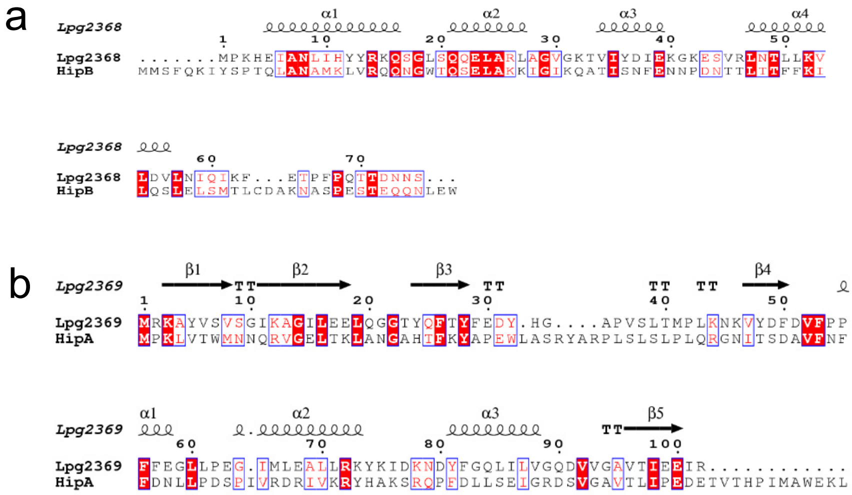

Supplementary Fig.3. Lpg2369 and Lpg2368 encoded by the genes in the same operon with *lpg2370* align with the N-terminal region of HipA and HipB from *E. coli* HipBA TA system, respectively. (**a**) Sequence alignment of Lpg2368 and the *E*. *coli* HipB antitoxin. (**b**) Sequence alignment of Lpg2369 and the N-terminus of *E*. *coli* HipA. The alignments were generated in ClustalW (https://www.genome.jp/tools-bin/clustalw) and visualized using ESPript (espript.ibcp.fr/ESPript/ESPript/). Strictly conserved residues are boxed and highlighted in white on red background, whereas highly conserved residues are boxed and highlighted in red on white background.

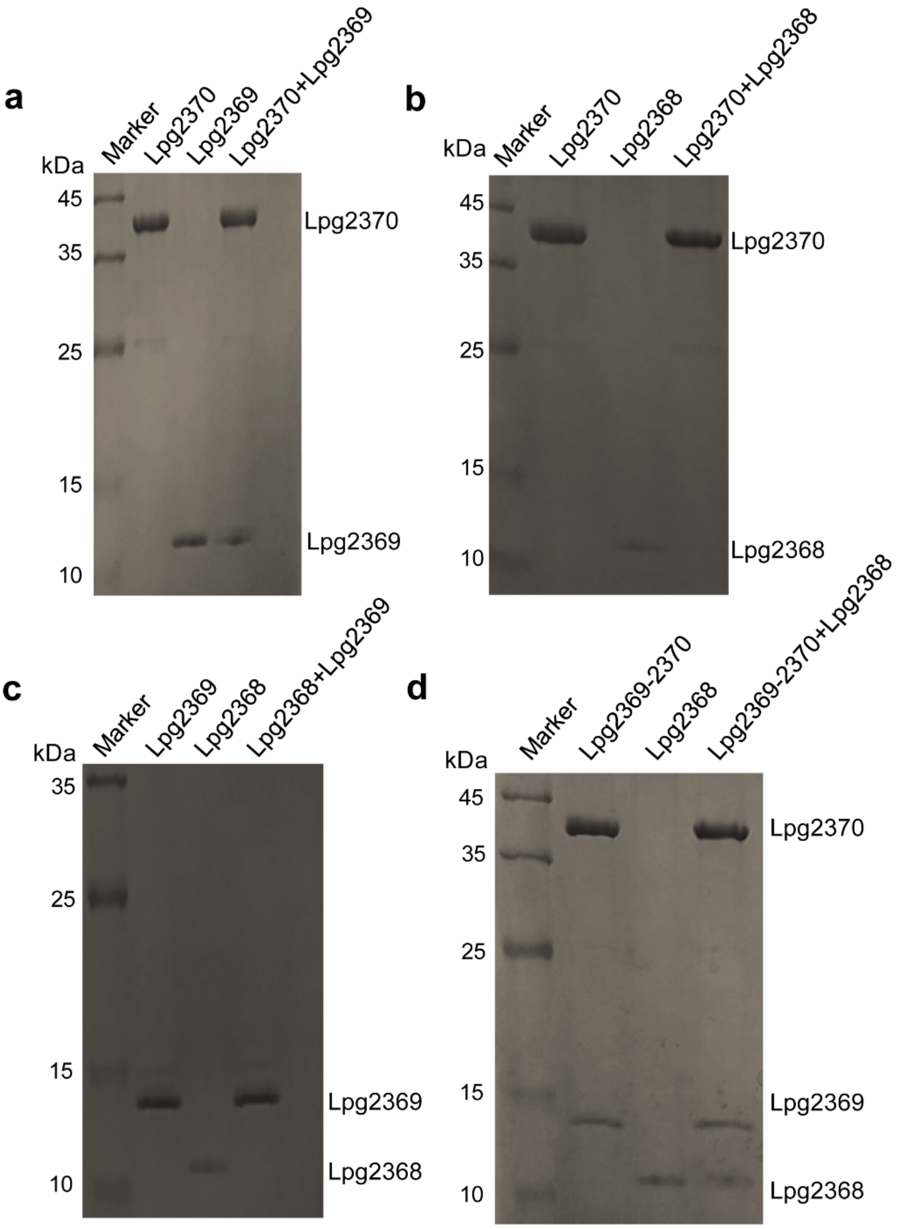

Supplementary Fig.4. Pull-down assays analyzing the interactions between Lpg2368, Lpg2370, and Lpg2369. (**a**) Pull-down assays confirm interaction between Lpg2369 and Lpg2370. (**b**) No direct interaction between Lpg2370 and Lpg2368 was detected. In (a) and (b), Lpg2370 with a C-terminal 6×His-tag, untagged Lpg2369 or untagged Lpg2368 were incubated with Ni-agarose beads for 30 min and then washed twice with buffer containing 20 mM Tris-HCl (pH 8.0) and 150 mM NaCl. Afterwards, the proteins were eluted using buffer containing 20 mM Tris-HCl (pH 8.0), 150 mM NaCl, and 300 mM imidazole. (**c**) Pull-down assays performed using Lpg2369 carrying C-terminal 6×His-tag and untagged Lpg2368 using a similar procedure as (a) and (b) suggest that there is no interaction between Lpg2369 and Lpg2368. (**d**) Pull-down assays performed with 6×His-tagged Lpg2369-Lpg2370 complex and untagged Lpg2368 using

a similar procedure as (a) and (b) demonstrate that Lpg2368 can bind to Lpg2369-Lpg2370 *in vitro*. All presented results are from one representative experiment done in triplicate.

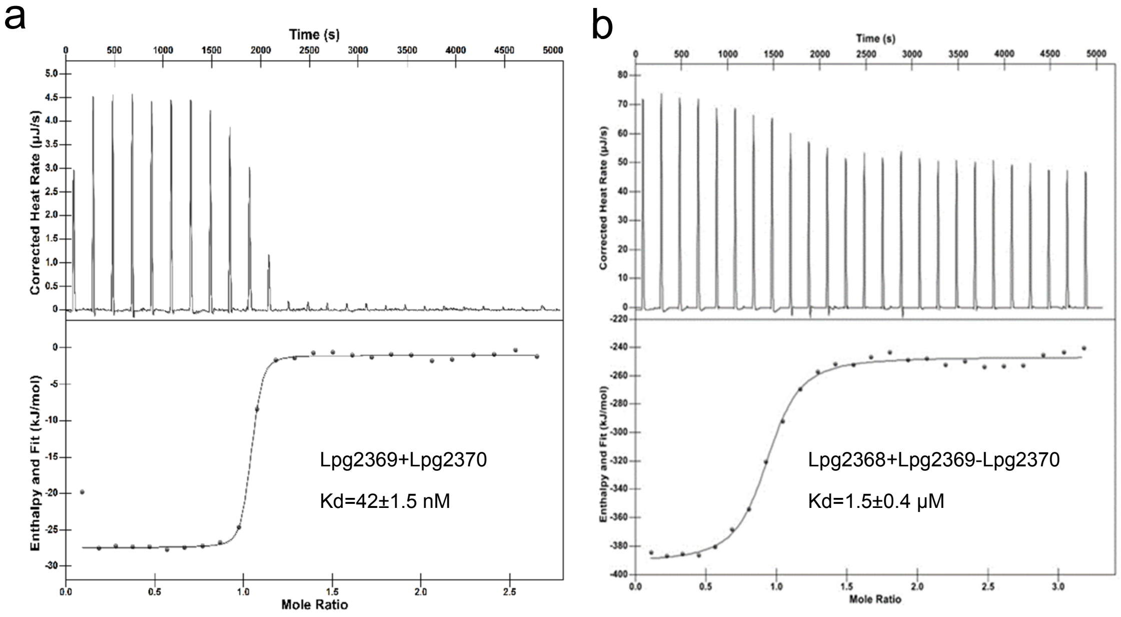

Supplementary Fig.5. Analysis of the interaction between Lpg2370 and Lpg2369, Lpg2370-Lpg2369 and Lpg2368 *in vitro*. (**a**) Binding of Lpg2369 and Lpg2370 monitored by ITC. The upper panel shows the original titration traces. The results revealed that Lpg2370 and Lpg2369 bind at high affinity (40 nM). The presented data is from a single ITC experiment. (**b**) The binding affinity of the co-purified Lpg2370-Lpg2369 with Lpg2368 measured by ITC. Lpg2368 was used to titrate the co-expressed Lpg2369-Lpg2370 in 20 mM Tris (pH 8.0), 150 mM NaCl. The dissociation constant (Kd) between Lpg2368 and the Lpg2370-Lpg2369 binary complex was determined at 1.5 $\pm$ 0.4 µM.

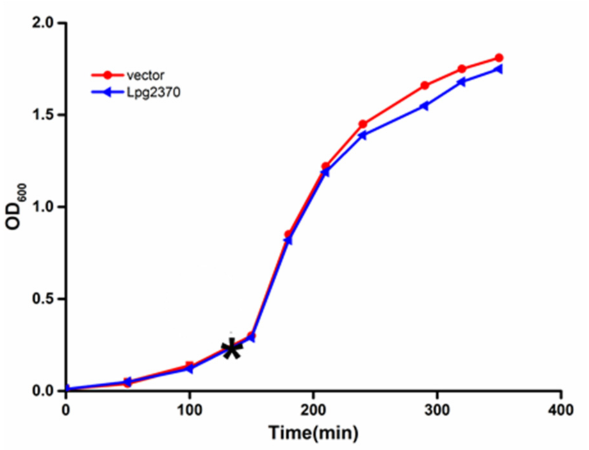

Supplementary Fig.6. Lpg2370 is not toxic to *E. coli*. Growth curve of *E. coli* expressing Lpg2370. The results shows that overexpression Lpg2370 does not cause growth arrest in OD_{600} when Lpg2370 was induced. the asterisk indicated the time point at which the expression of Lpg2370 was induced. The results are from one representative experiment done in triplicate.

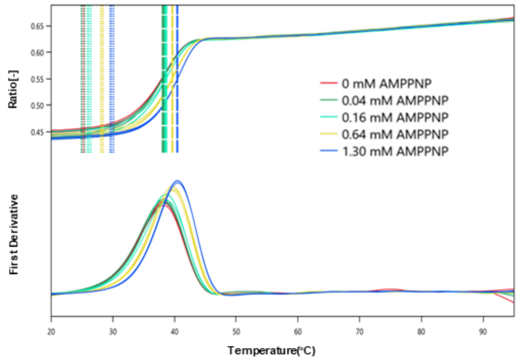

Supplementary Fig.7. The ATP binding ability of $HipT_{Lp}$ was abolished upon $HipS_{Lp}$ binding. Thermal shift assays of $HipT_{Lp}$ with AMP-PNP. $HipT_{Lp}$ was incubated with AMP-PNP at different concentrations as shown in the right side of the panel. The results show that the thermal stability of $HipT_{Lp}$ increases with the increase of AMP-PNP concentration.

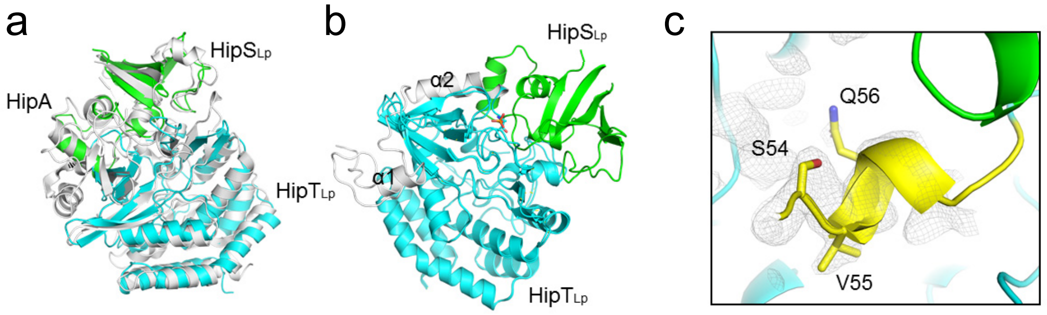

Supplementary Fig.8. Structural comparison of the apo HipT$_{Lp}$, HipT$_{Lp}$-HipS$_{Lp}$ and *E. coli* HipA. (**a**) Structural alignment of HipT$_{Lp}$-HipS$_{Lp}$ and *E. coli* HipA. HipT$_{Lp}$ and HipS$_{Lp}$ are colored cyan and green, respectively, and *E. coli* HipA is colored white. (**b**) Structural comparison of the apo- and HipS$_{Lp}$-bound HipT$_{Lp}$. HipT$_{Lp}$ and HipS$_{Lp}$ are colored cyan and green, respectively. The missing α1–2 in HipT$_{Lp}$ of the HipT$_{Lp}$-HipS$_{Lp}$ is colored white. (**c**) The residue S54 in not phosphorylated in the HipT$_{Lp}$-HipS$_{Lp}$ complex, revealed by the 2Fo-Fc omit map contoured at the 1.0 σ level.

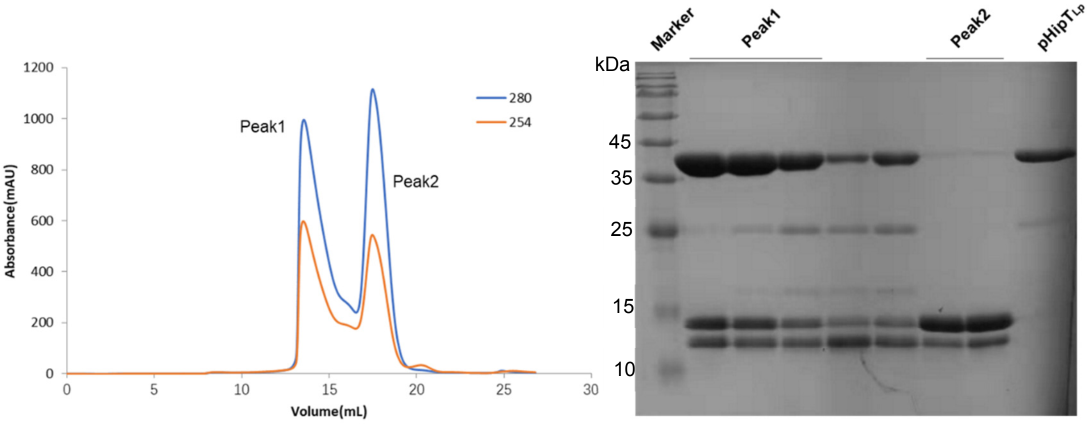

Supplementary Fig.9. Phosphorylation of S54 in HipT$_{Lp}$ does not affect the formation of the HipBST$_{Lp}$ heterotrimer. Left: size-exclusion chromatography analysis of the purified pHipT$_{Lp}$, HipS$_{Lp}$, and HipB$_{Lp}$ incubated at a 1:1.2:1.2 ratio. Right: SDS-PAGE analysis of the corresponding peak fractions suggests that they pHipT$_{Lp}$, HipS$_{Lp}$, and HipB$_{Lp}$ can form a heterotrimer (Peak 1). Peak 2 represents the mixture of excessive HipS$_{Lp}$, and HipB$_{Lp}$. The results are from one representative experiment performed in triplicate.

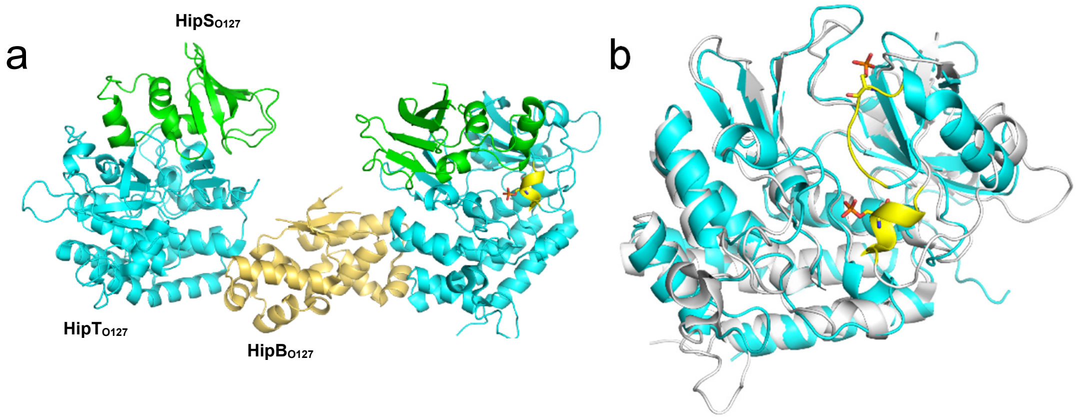

Supplementary Fig.10. Superimposition of pHipT$_{Lp}$ structure with HipT$_{O127}$ in the HipBST$_{O127}$ complex. (**a**) The overall structures of HipBST$_{O127}$ (PDB ID: 7AB3), HipT$_{O127}$, HipS$_{O127}$, and HipB$_{O127}$ are colored cyan, green, and yellow, respectively. (**b**) Loop-to helix-transition is also observed in the HipBST$_{O127}$. HipT$_{Lp}$ and HipT $_{O127}$ are colored cyan and grey, respectively. The P-loops of HipT$_{Lp}$ and the induced helix of HipT$_{O127}$ are colored yellow.

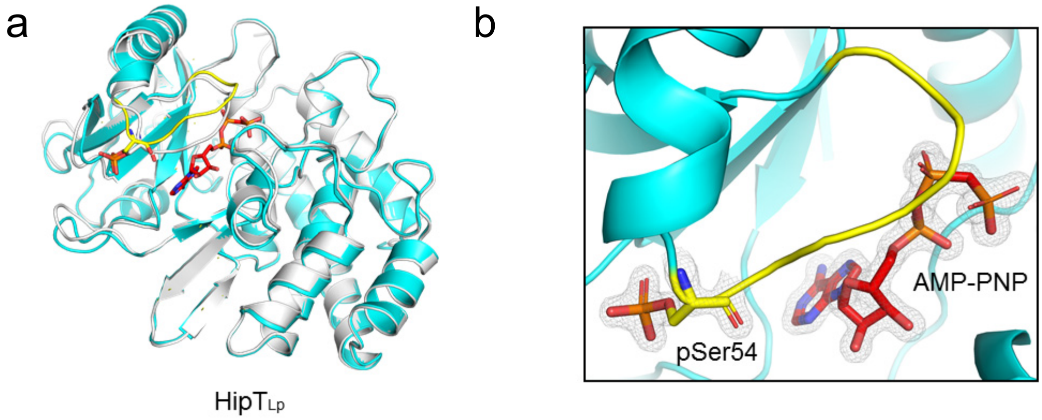

Supplementary Fig.11. Structural comparison of apo pHipT$_{Lp}$ and pHipT$_{Lp}$-AMP-PNP complex. (**a**) Structural alignment of the apo pHipT$_{Lp}$ with HipT$_{Lp}$-AMP-PNP complex. Apo pHipT$_{Lp}$ is colored white, the P-loop is colored yellow, and AMP-PNP is shown as red sticks. (**b**) Close-up view of the AMP-PNP. The 2Fo-Fc omit map of ATP was contoured at the 1.0 σ level.

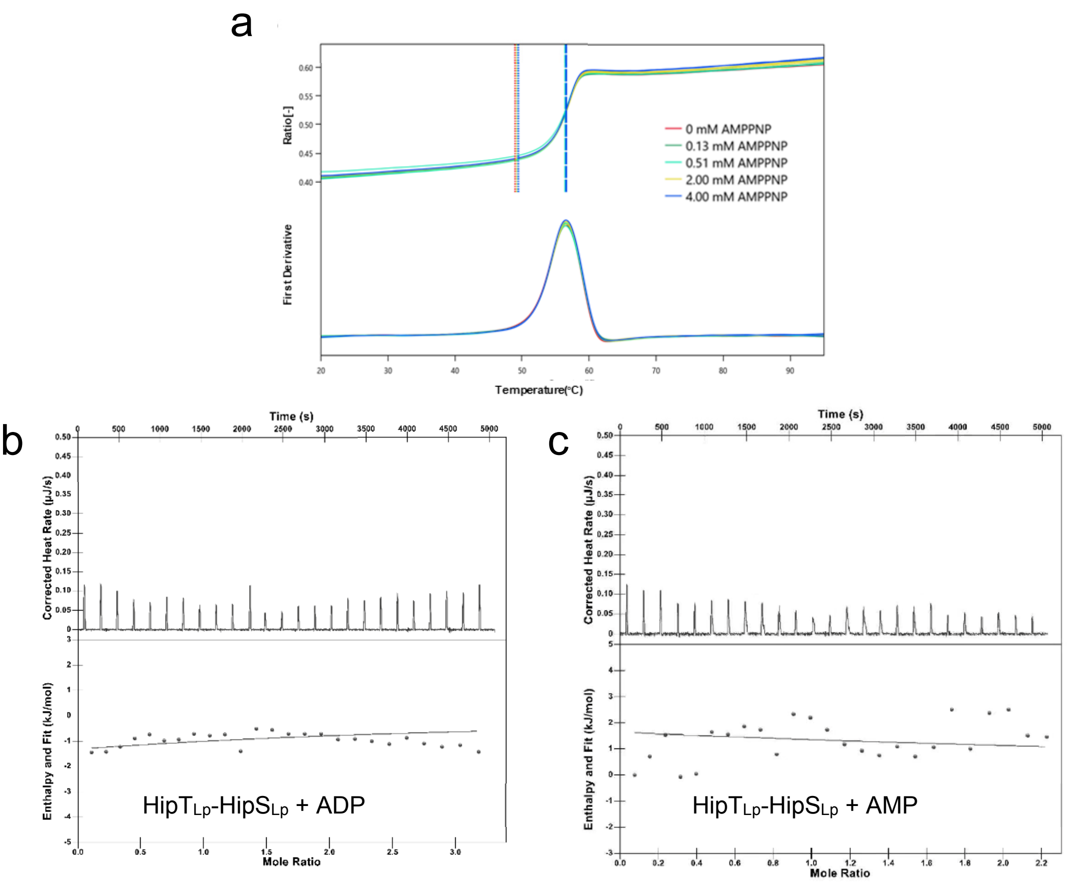

Supplementary Fig.12. The ATP binding ability of HipT$_{Lp}$ is abolished upon HipS$_{Lp}$ binding. (**a**) Thermal shift assays of HipT$_{Lp}$-HipS$_{Lp}$ with AMP-PNP, which was incubated with AMP-PNP at different gradient concentrations, revealing that the thermal stability of HipT$_{Lp}$-HipS$_{Lp}$ does not change with the increase of AMP-PNP concentration. (**b**) ITC measurement of the binding affinity between HipT$_{Lp}$-HipS$_{Lp}$ and ADP. (**c**) ITC measurement of the binding affinity between HipT$_{Lp}$-HipS$_{Lp}$ and AMP.

Supplementary Table 1. DALI search results against the PDB using the $HipT_{Lp}$ structure as query item.

No	PDB ID	Z scores	RMSD (Å)	Identity (%)	Description
1	4PU5	26.0	2.5	24	HipA family toxin-antitoxin system
2	3AKK	18.3	3.2	20	CtkA family kinase
3	5I0N	12.0	3.2	13	phosphatidylinositol 4 kinase
4	5WRR	10.7	3.7	13	FAM20A pseudokinase
5	5JDA	10.7	3.8	13	*Bacillus cereus* CotH kinase
6	1CJA	10.6	4.0	13	actin fragmin kinase
7	4YKN	10.3	4.3	8	PI3K lipid kinase
8	4D0L	9.9	4.1	9	PI4K kinase
9	6BQ1	9.1	4.4	11	PI4K kinase
10	3JBZ	8.9	4.5	9	ATM kinase
11	6SL1	8.8	4.6	9	Tel1 kinase
12	1HE8	8.8	4.4	9	PI3K kinase

Supplementary Table 2. Oligonucleotides used for plasmid construction and DNA sequencing in this study. F and R represent forward and the reverse primers, respectively.

Description	Sequence (5'→3')
**Primers for characterizing TA systems**	
Δ3(Δlpg2368-lpg2370)-A1	CGCGGATCCATAGGGAACACCACC
Δ3(Δlpg2368-lpg2370)-A2	TCCTCAATAGTCACTTTCGTGTTTGGGCAT
Δ3(Δlpg2368-lpg2370)-B1	ATGCCCAAACACGAAAGTGACTATTGAGGA
Δ3(Δlpg2368-lpg2370)-B2	CGCGTCGACTGATTGAGACTGCGG
Lpg2368-Lpg2369-Lpg2370-F1	CGCGGATCCATAGGGAACACCACC
Lpg2368-Lpg2369-Lpg2370-F2	GCTTGCATCGTTGCTTTCGTGTTTGGGCAT
Lpg2368-Lpg2369-Lpg2370-R1	ATGCCCAAACACGAAAGCAACGATGCAAGC
Lpg2368-Lpg2369-Lpg2370-R2	CGCGTCGACAGGGGCCAGATATAC
Lpg2370-F	CTGGGATCCATGAAACACTGCCCTATTA
Lpg2370-R	CTGGTCGACTTAATCAAAAAAATTTAATCGC
Lpg2369-F	CTGGGATCCATGAGAAAAGCATACGTATC
Lpg2369-R	CTGGTCGACTTATCATCTTATTTCCTCAATA
Lpg2368-F	CTGGGATCCATGCCCAAACACGAAAT
Lpg2368-R	CTGGTCGACTTATCATGAATTATTATCCGTT
**Primers for site-directed mutagenesis using fusion PCR**	
HipT$_{Lp}$^{Q78A}-F	AAAGAAGGCTGTTTTGAAATCGTGGATGCATATGGTCAGTATATTTTAAAACCA
HipT$_{Lp}$^{Q78A}-R	ATGCATCCACGATTTCAAAACAGCCTTCTTTAATCTTTAGTTTTGCGCTTAG
HipT$_{Lp}$^{Y79A}-F	GAAGGCTGTTTTGAAATCGTGGATCAAGCTGGTCAGTATATTTTAAAACCACAA
HipT$_{Lp}$^{Y79A}-R	CAGCTTGATCCACGATTTCAAAACAGCCTTCTTTAATCTTTAGTTTTGCGCT
HipT$_{Lp}$^{D133A}-F	AGTTTAACCTACTTCATTAAACGCTTTGCTAGAATAGGCCATAATAAAAAGTTA
HipT$_{Lp}$^{D133A}-R	TAGCAAAGCGTTTAATGAAGTAGGTTAAACTGTTGTCTTTAGAATAAACCAA
HipT$_{Lp}$^{R134A}-F	TTAACCTACTTCATTAAACGCTTTGATGCAATAGGCCATAATAAAAAGTTAGCT

$HipT_{Lp}^{R134A}$-R	TTGCATCAAAGCGTTTAATGAAGTAGGTTAAACTGTTGTCTTTAGAATAAAC
$HipT_{Lp}^{E144A}$-F	GGCCATAATAAAAAGTTAGCTTTAGCAGATTTTGCACAGCTATCAGGTGAA
$HipT_{Lp}^{E144A}$-R	CTGCTAAAGCTAACTTTTTATTATGGCCTATTCTATCAAAGCGTTTAATGAA
$HipT_{Lp}^{R154A}$-F	TTTGCACAGCTATCAGGTGAAGATGCACATACAAAATATAAAAGTTCTATG
$HipT_{Lp}^{R154A}$-R	GTGCATCTTCACCTGATAGCTGTGCAAAATCTTCTAAAGCTAACTTTTTATT
$HipT_{Lp}^{K157A}$-F	CAGCTATCAGGTGAAGATCGACATACAGCATATAAAAGTTCTATGGAAAAAGTAATT
$HipT_{Lp}^{K157A}$-R	ATGCTGTATGTCGATCTTCACCTGATAGCTGTGCAAAATCTTCTAAAGCTAA
$HipT_{Lp}^{K201A}$-F	GTCGGTAATGAAGACATGCATCTAGCAAACTTTTCCTTAATTACGAAGGAT
$HipT_{Lp}^{K201A}$-R	TTGCTAGATGCATGTCTTCATTACCGACCAAGAAGTTAAACAACGTCAACTT
$HipT_{O127}^{S57A}$-F	CAGCAGAAAGGCATGGCCATTAGCGGTTACCAG
$HipT_{O127}^{S57A}$-R	TGGCCATGCCTTTCTGCTGGCG
$HipT_{O127}^{S57D}$-F	CAGCAGAAAGGCATGGACATTAGCGGTTACCAG
$HipT_{O127}^{S57D}$-R	TGTCCATGCCTTTCTGCTGGCG
$HipT_{O127}^{H212A}$-F	CTGGGTAACAACGATATGGCTCTGCGTAACTTCGGTCTG
$HipT_{O127}^{H212A}$-R	GAGCCATATCGTTGTTACCCAGCAGCCATGCGTAAAC
$HipT_{O127}^{N215A}$-F	AACGATATGCATCTGCGTGCCTTCGGTCTGGTGTACTCT
$HipT_{O127}^{N215A}$-R	AGCTACGCAGATGCATATCGTTGTTACCCAGCAGCCA
$HipT_{O127}^{D233A}$-F	GCCCCGGTTTACGCTTTCGTGAGCGTT
$HipT_{O127}^{D233A}$-R	AAGCGTAAACCGGGGCCAGTGCCGG
**Primers for protein expression and purification**	
pET21a-$HipT_{Lp}$-F	TACTTCCAATCCAATGCCATGAAACACTGCCCTATTACTTAT
pET21a-$HipT_{Lp}$-R	TTATCCACTTCCAATGTTATTAATCAAAAAAATTTAATCGCTTGCA
pET21a-$HipS_{Lp}$-F	TACTTCCAATCCAATGCCATGAGAAAAGCATACGTATCG
pET21a-$HipS_{Lp}$-R	TTATCCACTTCCAATGTTATCATCTTATTTCCTCAATAGTCACTGC
pET21a-$HipB_{Lp}$-F	TACTTCCAATCCAATGCCATGCCCAAACACGAAATTG
pET21a-$HipB_{Lp}$-R	TTATCCACTTCCAATGTTATCATGAATTATTATCCGTTGTTTG
pET21a-$HipS_{Lp}$-$HipT_{Lp}$-F	GAGGAAATAAGATGATTTGTTTAACTTTAAGAAGGAGATATACATATGAAACACTGC CCTATTACTTATAAACACTGC
pET21a-$HipS_{Lp}$-$HipT_{Lp}$-R	TCATCTTATTTCCTCAATAGTCACTGC

pET21a-HipT$_{O127}$-F	TACTTCCAATCCAATGCCATGGCGAACTGTCGTATTCT
pET21a-HipT$_{O127}$-R	TTATCCACTTCCAATGTTACAGCAGGCCCAGACG
**Primers for DNA sequencing**	
pET21a-f (T7-F)	TAATACGACTCACTATAGGG
pET21a-r (T7-R)	TATGCTAGTTATTGCTCAG
